# Transcriptome analysis identifies genes related to the waxy coating on blueberry fruit in two northern-adapted rabbiteye breeding populations

**DOI:** 10.1186/s12870-019-2073-7

**Published:** 2019-10-31

**Authors:** Xinpeng Qi, Elizabeth L. Ogden, Jose V. Die, Mark K. Ehlenfeldt, James J. Polashock, Omar Darwish, Nadim Alkharouf, L. Jeannine Rowland

**Affiliations:** 1 USDA-ARS, BARC-West, Genetic Improvement of Fruits and Vegetables Laboratory, Beltsville, MD 20705 USA; 2 Departmento de Genetica, University of Córdoba Campus Rabanales, Blg. C5, 14071 Córdoba, Spain; 3 USDA-ARS, Genetic Improvement of Fruits and Vegetables Laboratory, at Rutgers University P.E. Marucci Center for Blueberry and Cranberry Research and Extension, Chatsworth, NJ 08019 USA; 4 Department of Mathematics and Computer Science, Texas Woman’s University, Denton, TX 76204 USA; 5 Department of Computer and Information Sciences, Towson University, Towson, MD 21252 USA

**Keywords:** Bulked segregant analysis, Differential expression, Fruit wax, RNA-seq, *Vaccinium* spp.

## Abstract

**Background:**

Blueberry is of high economic value. Most blueberry varieties selected for the fresh market have an appealing light blue coating or “bloom” on the fruit due to the presence of a visible heavy epicuticular wax layer. This waxy layer also serves as natural defense against fruit desiccation and deterioration.

**Results:**

In this study, we attempted to identify gene(s) whose expression is related to the protective waxy coating on blueberry fruit utilizing two unique germplasm populations that segregate for the waxy layer. We bulked RNA from waxy and non-waxy blueberry progenies from the two northern-adapted rabbiteye hybrid breeding populations (‘Nocturne’ x T 300 and ‘Nocturne’ x US 1212), and generated 316.85 million RNA-seq reads. We de novo assembled this data set integrated with other publicly available RNA-seq data and trimmed the assembly into a 91,861 blueberry unigene collection. All unigenes were functionally annotated, resulting in 79 genes potentially related to wax accumulation. We compared the expression pattern of waxy and non-waxy progenies using edgeR and identified overall 1125 genes in the T 300 population and 2864 genes in the US 1212 population with at least a two-fold expression difference. After validating differential expression of several genes by RT-qPCR experiments, a candidate gene, *FatB*, which encodes acyl-[acyl-carrier-protein] hydrolase, emerged whose expression was closely linked to the segregation of the waxy coating in our populations. This gene was expressed at more than a five-fold higher level in waxy than non-waxy plants of both populations. We amplified and sequenced the cDNA for this gene from three waxy plants of each population, but were unable to amplify the cDNA from three non-waxy plants that were tested from each population. We aligned the *Vaccinium* deduced FATB protein sequence to FATB protein sequences from other plant species. Within the PF01643 domain, which gives FATB its catalytic function, 80.08% of the amino acids were identical or had conservative replacements between the blueberry and the *Cucumis melo* sequence (XP_008467164). We then amplified and sequenced a large portion of the *FatB* gene itself from waxy and non-waxy individuals of both populations. Alignment of the cDNA and gDNA sequences revealed that the blueberry *FatB* gene consists of six exons and five introns. Although we did not sequence through two very large introns, a comparison of the exon sequences found no significant sequence differences between the waxy and non-waxy plants. This suggests that another gene, which regulates or somehow affects *FatB* expression, must be segregating in the populations.

**Conclusions:**

This study is helping to achieve a greater understanding of epicuticular wax biosynthesis in blueberry. In addition, the blueberry unigene collection should facilitate functional annotation of the coming chromosomal level blueberry genome.

## Background

Cultivation of blueberries is rapidly expanding worldwide. Production in the United States, the largest producer of blueberries, increased by 33% from 2010 to 2015 and by 119% from 2005 to 2015 (United States Department of Agriculture-National Agricultural Statistics Service (USDA-NASS)). Production worldwide also grew sharply in recent years, by 58% from 2009 to 2014, and now exceeds 525,000 metric tons (United Nations, Food and Agriculture Organization). Consumption of blueberries has increased as well, likely driven by greater awareness of the many health benefits of anthocyanins. Blueberries have been shown to be one of the richest sources of anthocyanins and antioxidants of all fresh fruits and vegetables [1]. In the U.S., per capita annual consumption of blueberries rose from 0.6 lb. in 2000 to 1.5 lb. in 2010 [2].

There are three major species of blueberries grown commercially, the tetraploid highbush blueberry (*Vaccinium corymbosum* and hybrids thereof), the hexaploid rabbiteye blueberry (*V. virgatum*), and the wild tetraploid lowbush blueberry (*V. angustifolium*). Blueberry breeding efforts have focused on traits for broad climatic adaptation, season extension, disease and pest resistance, mechanical handling tolerance, and high fruit quality [3]. Fruit quality includes many attributes such as good flavor, large size, firmness, and light blue color, among others. The color of blueberries is due to the presence of anthocyanins within the fruit and to a cuticular waxy layer on the outside of the fruit. This waxy coating or “bloom” gives the desirable light blue color. Besides being more visually appealing than black-colored fruit, the waxy coating retards fruit desiccation and deterioration in storage [4].

The cuticle of land plants, a hydrophobic layer covering the aerial surfaces of all organs including fruit, is comprised of cutin and cuticular waxes. It is thought that the cuticle acts as the first protective barrier against non-stomatal water loss, reduces effects of biotic/abiotic stress, and changes light reflectance [5]. The cuticle is made up of three layers: the innermost layer or the cuticular layer (CL) composed of cutin, intracuticular waxes, and polysaccharides; the cuticle proper (CP) made up of epicuticular waxes, intracuticular waxes, and cutin; and finally the outermost layer comprised of epicuticular waxes (EW) [6]. Cuticular waxes are comprised of a mixture of very-long-chain (VLC) aliphatic compounds, triterpenoids, and other metabolites like sterols and flavonoids. Aliphatic acyl chains of waxes are derived from VLC-fatty acids (VLCFAs) by way of two distinct biosynthetic pathways, the alcohol-forming pathway, yielding primary alcohols and alkyl esters, and the alkane-forming pathway, yielding aldehydes, alkanes, secondary alcohols, and ketones [6].

The chemical composition of cuticular wax on blueberry fruit has been recently characterized using gas chromatography-mass spectrometry and scanning electron microscopy. Triterpenoids and β-diketones were found to be the predominant compounds, accounting for 64.2 and 16.4%, respectively, of the total waxes [7]. From studies on wheat and barley, it is thought that triterpenoids mainly form amorphous wax both in the intracuticular layer and the epicuticular layer, while β-diketones mainly form crystalline wax in the epicuticular layer, which gives the visible glaucous appearance [8, 9].

In this study, we sought to identify gene(s) whose expression is related to the protective waxy coating on blueberry fruit utilizing two unique germplasm populations. These populations resulted from crosses made in our blueberry breeding program (northern-adapted rabbiteye hybrid breeding populations) that segregate visibly for the presence/absence of the waxy coating. RNA-seq was performed on bulked RNA from progeny that have the waxy coating and progeny that do not. Of the genes that were differentially expressed between the two bulks, several candidates were selected and their expression was tested on the individual plants that comprised the original bulks by real-time qPCR. From these analyses, our best candidate gene(s), differentially expressed in both populations and related to wax biosynthesis or transport, were identified. In this study, we also generated the most comprehensive blueberry transcriptome assembly ever reported, which should benefit the whole community of blueberry researchers and facilitate annotation of the blueberry genome.

## Results

### Blueberry transcriptome sequencing and assembly

**Fig. 1:**
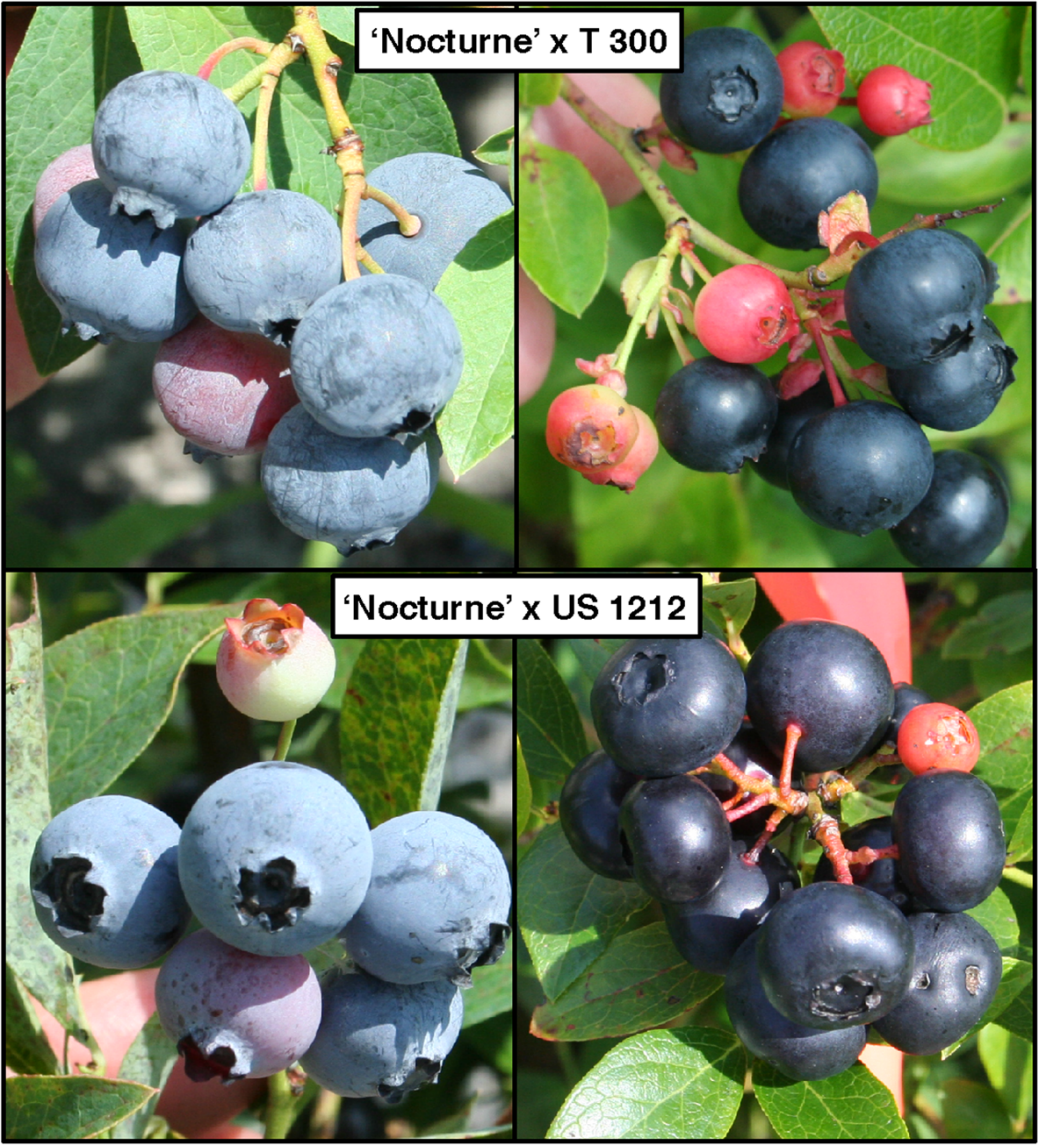
Blueberry fruit of individual plants from two blueberry populations segregating for the presence of wax. Top panel: representative plants of ‘Nocturne’ x T 300 population with waxy (left) and non-waxy (right) coating on fruit; bottom panel: representative plants of ‘Nocturne’ x US 1212 population with waxy (left) and non-waxy (right) coating on fruit

In an attempt to identify gene(s) related to the protective waxy coating on blueberry fruit, two unique germplasm populations were utilized. These populations resulted from crosses made in our northern rabbiteye breeding program that segregate visibly for the presence/absence of the waxy coating (Fig. 1). Segregation ratios for the waxy coating on fruit of the populations are described in Table 1. RNA-seq was performed on RNA extracted from bulked fruit tissue of progeny that have the waxy coating and progeny that do not. The four RNA-seq libraries (from the waxy and non-waxy bulks of the two populations) yielded a total of 316.85 million Illumina paired-end 100 bp reads (Additional file 1: Table S1).

**Table 1:** Segregation of the waxy coating on fruit in the two blueberry populations

Number of plants with a rating of:							
Population	0	1	2	3	4	5	Segregation ratio^a^
‘Nocturne’ x T 300	17	4	6	3	2	0	21:11^b^
‘Nocturne’ x US 1212	15	5	6	1	7	2	20:16

Plants were given a score of 0–5 based on the amount of wax that was visually apparent on the fruit. Scores of 0 or 1 indicated no or little wax, scores of 2 or 3 indicated presence of a medium amount of wax, and scores of 4 or 5 indicated presence of a heavy waxy coating

^a^Segregation ratio here is the number of plants with a score of 0–1: number of plants with a score of 2–5

^b^One plant in the ‘Nocturne’ x T 300 population could not be scored because it did not produce fruit

In addition to the reads generated from this study, another 376.77 million paired-end and 485.40 million single-end Illumina reads previously generated from blueberry were downloaded from the SRA of NCBI. After quality trimming, these 90.77 Gbp of clean reads were mapped to the most recent version of the blueberry genome assembly available (Robert Reid, UNC, and Allan Brown, IITA, personal communication). The libraries from our study had the top mapping rate (average 91%) of all analyzed. Most of the other libraries also had good mapping rates (average 74%), except for the library SRR1187674, which had a mapping rate too low to be considered as blueberry transcriptome data (Additional file 1: Table S2). After excluding this library, the remaining 87.34 Gbp of clean RNA-seq reads were used for transcriptome assembly.

**Fig. 2:**
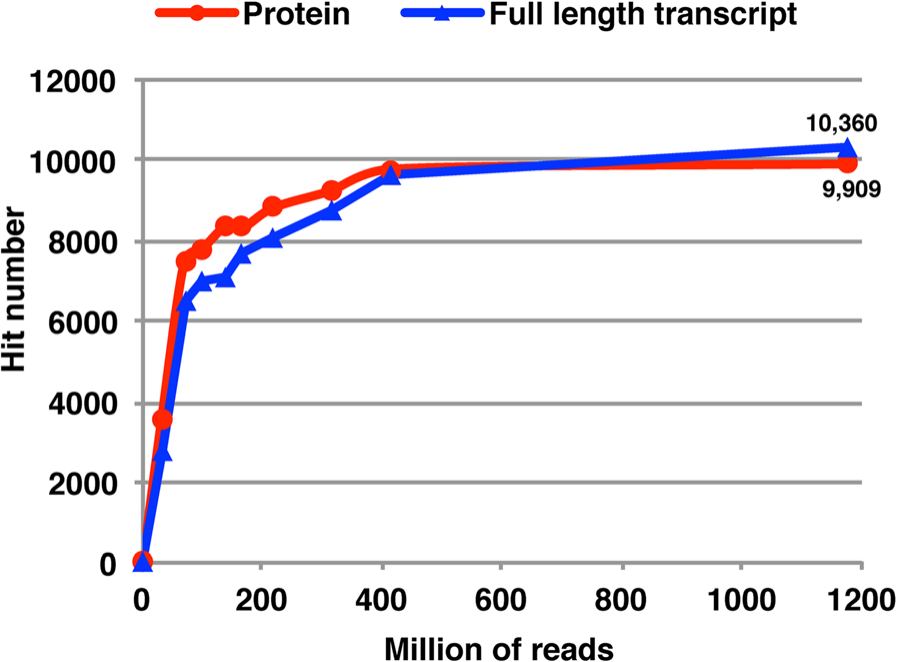
Blueberry transcriptome laddered de novo assembly based on different millions of RNA-seq reads

A laddered de novo assembly strategy was performed to evaluate whether this data set was adequate to give a comprehensive blueberry transcriptome assembly. A series of different millions of reads were randomly selected and de novo assembly was performed based on each data set. In terms of full-length transcript representation and protein hits to non-model organisms, more transcripts were captured with increasing read number until a plateau was reached at about 400 million reads (Fig. 2). We also compared contigs previously assembled by our laboratory using 454 sequences [10, 11]: as it turns out, 32,794 out of 37,524 (86.67%) 454 contigs had a hit to the new de novo assembly (Additional file 1: Table S3). Thus, we concluded that the total clean data set of ~ 1100 million reads used for the blueberry transcriptome assembly in this study was enough to give a saturated assembly.

We also performed a reference-based assembly based on this ~ 1100 million read data set, assembly workflow is shown in Additional file 1: Figure S1. When mapping reads back to the assembled transcripts, however, the reference-based assembly had a much lower mapping rate as compared to the de novo version (Additional file 1: Table S2). This could be due to the incompleteness of this version of the reference genome and/or mistakes in the reference genome annotation. Thus, we chose to use the de novo version of the transcript assembly for our study and only used the reference-based assembly as supportive evidence of transcript confidence.

### Blueberry unigene collection and functional annotation

A total of 251,974 Trinity ‘genes’ and 352,293 Trinity ‘transcripts’ were generated from the initial raw de novo assembly (Additional file 1: Table S4). This assembly had an average of 77.4% read-mapping-back rate (Additional file 1: Table S2, average of column 4). Assembled transcripts hit 9909 protein records in SwissProt database [12] and captured 10,360 full-length blueberry genes (Fig. 2). These results indicated the assembly was of good quality. However, after identifying coding regions by TransDecoder, 8037 transcripts had exactly the same coding sequences. We reduced this kind of redundancy by selecting the longest predicted CDS sequence for each of the Trinity ‘genes’ (Additional file 1: Figure S2). We further applied CD-HIT [13] to polish the selected assembly, which resulted in 91,861 candidate Trinity ‘genes’. We then used TGICL [14] to double check this candidate assembly. Only 460 clusters were found, which indicated low redundancy. Thus, the 91,861 candidate Trinity ‘gene’ set was considered a good, non-redundant blueberry unigene dataset (Table 2).

**Table 2:** Blueberry unigene statistics and functional annotation

Unigene	
Total number	91,861
N50 length	1144
Mean length	725.47
Total length	66,642,205
DataBase	Annotated (%)
SwissProt	32,586 (35.47)
Trembl	55,449 (60.36)
NCBI-Nr	56,175 (61.15)
PlantCyc	14,231 (15.49)
refPlant	39,913 (43.45)
KEGG	14,762 (16.07)
Total	56,696 (61.72)

All unigenes were then subjected to public protein database searches including SwissProt, TrEMBL, NCBI-Nr database, and refPlant for functional annotation. Enzyme records from pathway databases, PlantCyc and KEGG, were also used for gene function annotation. In total, 56,696 unigenes (61.72%) were annotated by at least one hit of public databases. NCBI-Nr database annotated the most unigenes. Among all refPlant annotated species, grape (*Vitis vinifera*) resulted in the most hits to the blueberry unigenes (Additional file 1: Figure S3). A total of 14,231 enzyme records from 847 PlantCyc metabolic pathways were assigned to the blueberry unigene data set.

### Blueberry unigenes annotated as potentially cuticular wax-related proteins

To better understand the genetic basis of plant cuticular wax deposition, we searched the literature and found 47 relevant references (Additional file 1: Table S5). From these sources, we collected 112 protein records encoded by 88 genes reported to be related to plant cuticular wax accumulation and incorporated 447 enzymes from the PlantCyc cuticular wax biosynthesis pathway, PWY-282, to establish our own wax database, which we call ‘waxybase’. We then applied stringent searching criteria between waxybase and the blueberry unigene data set for annotation. As a result, 79 blueberry genes were annotated by 46 waxybase proteins (Table 3). *Arabidopsis* and tomato (*Solanum lycopersicum*) provided the most reference records in this annotation. These wax-related blueberry genes were annotated as various key enzymes including biosynthesis genes CER1/3/6/9/10, regulatory factors MYB41/106, and wax secretion-related genes ABCG11/12/32 (Additional file 1: Table S6).

**Table 3:** Blueberry genes annotated by waxybase proteins

Function	Species	GeneName	Number of Homologous Blueberry Genes
Biosynthesis	*Arabidopsis thaliana*	CER6/9/10	8
		LACS1/2/4	5
		KCS1/2	5
		ACLA2	4
		PAS2	1
		FDH	1
		FATB	1
	*Solanum lycopersicum*	CER1/6	4
		FAR	4
		Cytochrome P450	1
	*Vitis vinifera*	CER1/3	3
		FAR	2
	*Solanum tuberosum*	Cytochrome P450	1
	*Medicago truncatula*	WXP1	1
	*Zea mays*	GL8b	1
	*Manihot esculenta*	FAR	1
	*Populus trichocarpa*	CER3	1
Cutin biosynthesis	*Arabidopsis thaliana*	LCR	5
		Homolog of CD1	2
		GPAT6	1
		BDG3	1
	*Solanum lycopersicum*	CD1	2
Regulation	*Arabidopsis thaliana*	MYB41/106	3
		RDR1	1
		CER7	1
	*Solanum lycopersicum*	SlTTS1	2
Cutin regulation	*Arabidopsis thaliana*	WIN1/ SHN1	1
Secretion	*Arabidopsis thaliana*	ABCG11/12/32	14
Enigmatic Factors	*Arabidopsis thaliana*	HTH	2

### Differentially expressed genes (DEGs) related to cuticular wax accumulation

**Fig. 3:**
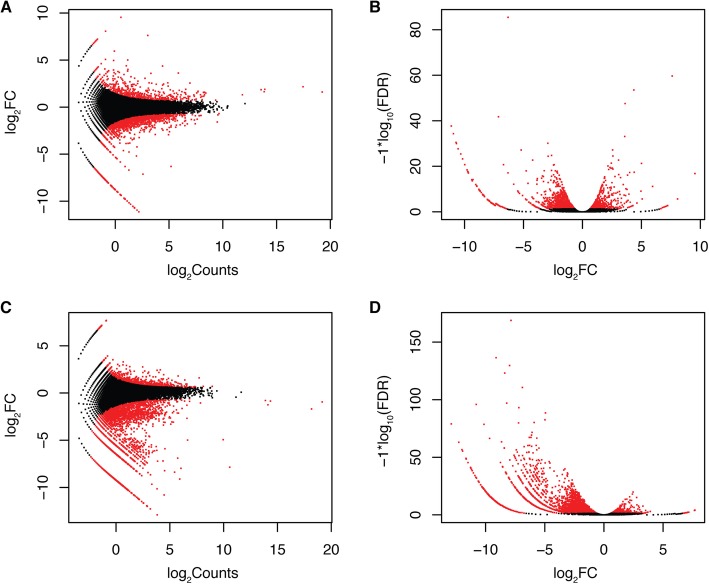
Expression profiles of waxy versus non-waxy blueberry genes based on fruit tissue expression data. **a** and **c**. MA plot (log_2_ fold change versus log_2_ counts) of all assembled genes in ‘Nocturne’ x T 300 and ‘Nocturne’ x US 1212 population; **b** and **d**. Volcano plot of log_10_ false discovery rate versus log_2_ fold change in ‘Nocturne’ x T 300 and ‘Nocturne’ x US 1212 populations. Differentially expressed genes with FDR < =0.05 are marked in red

**Fig. 4:**
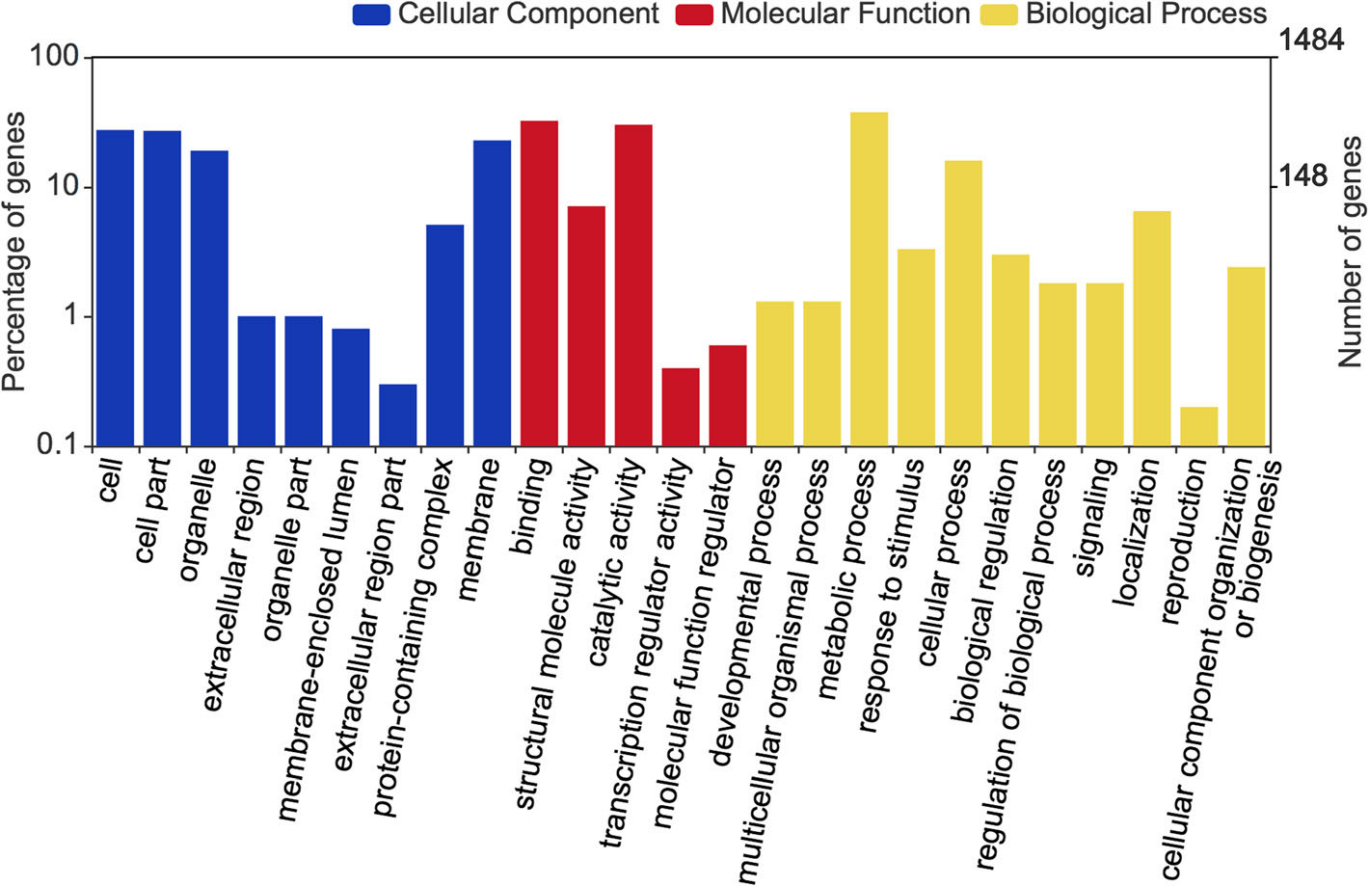
GO annotation of identified differentially expressed genes between waxy and non-waxy blueberry fruit tissues. X-axis shows GO terms; y-axis shows gene number and percentage in log scale

To uncover genes whose expression levels are related to segregation of the protective waxy coating on fruit observed in our unique blueberry populations, differential expression analysis was performed using edgeR [15]. We identified 1125 genes in the ‘Nocturne’ x T 300 population and 2864 genes in the ‘Nocturne’ x US 1212 population with at least a two-fold difference in expression level between the waxy and non-waxy libraries (Additional file 2: Table S7 and Additional file 3: Table S8). Interestingly, more genes had higher expression levels in the non-waxy blueberry libraries than in the waxy libraries of both populations (Fig. 3). A total of 3333 DEGs (96.47%) found hits with NCBI Nr database, and they were then mapped to Gene Ontology (GO) entries by BLAST2GO (Fig. 4). Twenty-five GO functional categories had hits including “membrane” and “protein-containing complex” functions. Four of the identified DEGs were annotated in waxybase as well, and these genes were selected, among others (described below), for RT-qPCR validation.

### Expression analysis of identified DEGs and other genes known to be involved in wax biosynthesis

We first combined edgeR predicted expression results together with functional annotation results, and selected 26 genes for RT-qPCR validation (Additional file 4: Table S9). Of these 26, four genes had predicted higher expression levels in the waxy bulks than the non-waxy bulks of both populations (Log_2_FC = 2, FDR = 0.001). Another four genes (the top four of a total of 85) had predicted higher expression levels in the non-waxy bulks than the waxy bulks of both populations (Log_2_FC = 2, FDR = 0.001). In addition, we chose seven of the top 12 genes in the ‘Nocturne’ x T 300 population and four of the top 10 genes in the ‘Nocturne’ x US 1212 population that were predicted to be expressed at higher levels in the waxy bulk than the non-waxy bulk of only that one population (Log_2_FC = 1, FDR = 0.005). Finally, of the genes selected for RT-qPCR, we included the only two genes that had higher predicted expression levels in the waxy bulk than in the non-waxy bulk of the ‘Nocturne’ x US 1212 population and had a hit with the waxy database (Log_2_FC = 1, FDR = 0.005). We also chose the top five genes (out of a total of eight) that had higher predicted expression levels in the non-waxy bulk than in the waxy bulk of the ‘Nocturne’ x US 1212 population and had a hit with the waxy database (Log_2_FC = 1, FDR = 0.005). One of these was also predicted to have higher expression levels in the non-waxy bulk than in the waxy bulk of the ‘Nocturne’ x T 300 population. No other genes that were predicted to be differentially expressed in the ‘Nocturne’ x T 300 population had hits with the waxybase (Log_2_FC = 1, FDR = 0.005).

**Fig. 5:**
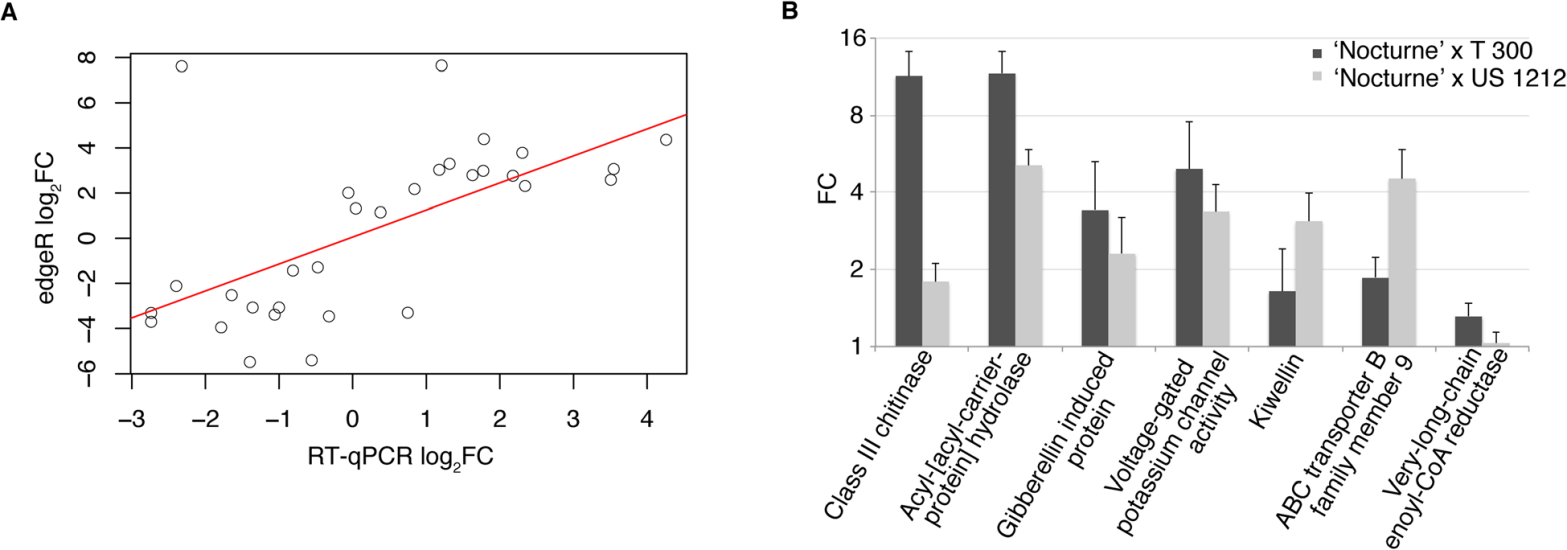
RT-qPCR validation experiment of candidate DEGs. **a**. Linear regression of log_2_ fold change between edgeR predicted expression and RT-qPCR expression. **b**. RT-qPCR results of various candidate genes with different expression in waxy bulks and non-waxy bulks in either one or both populations. Results presented are averages (means) of RT-qPCR data from individual plants that comprised each bulk

**Fig. 6:**
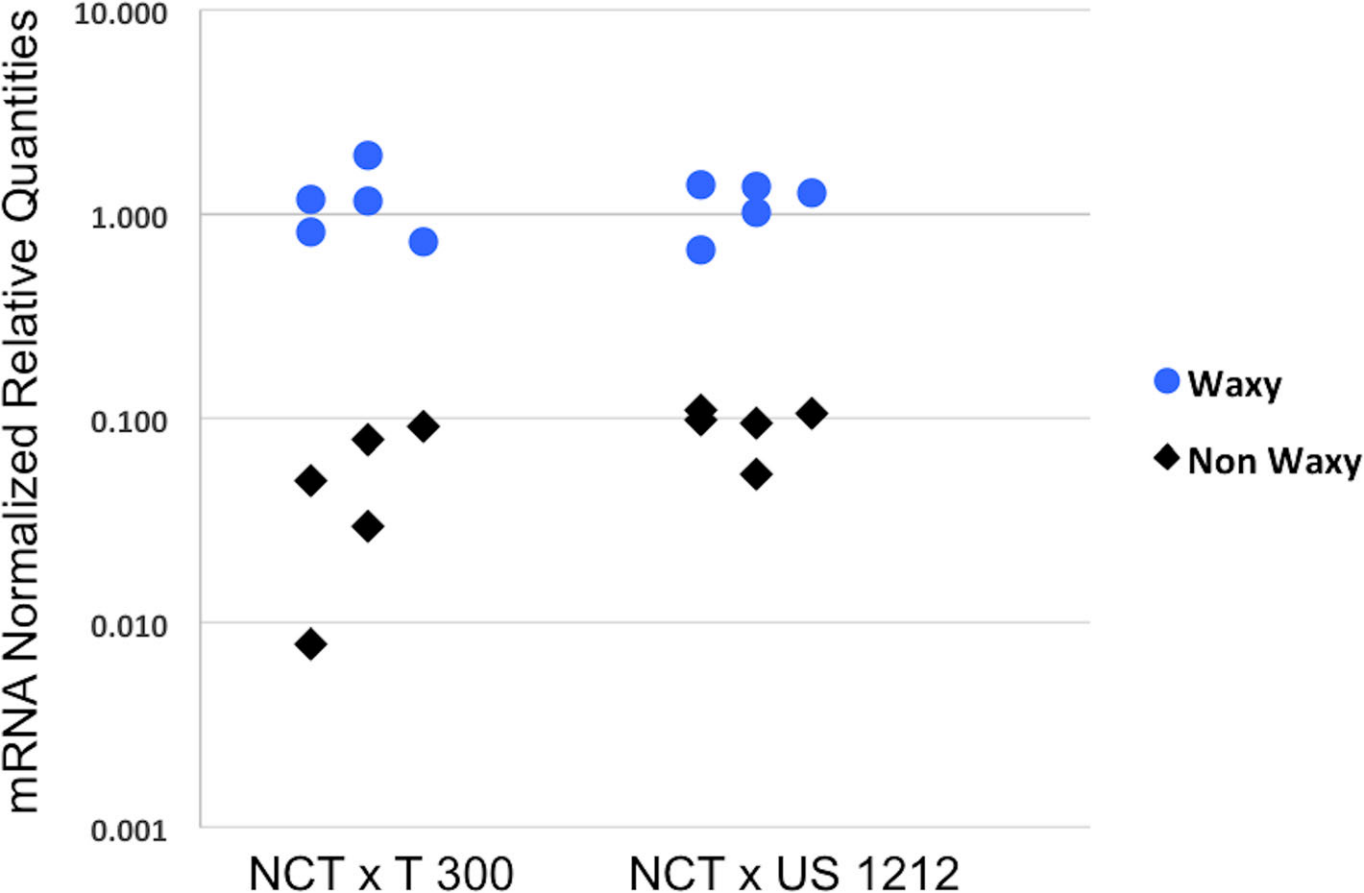
RT-qPCR results of the differentially expressed acyl-[acyl-carrier-protein] hydrolase homolog, or *FatB* gene, from fruit tissue of five individual plants that comprised each of the waxy and non-waxy bulks from both populations, ‘Nocturne’ x T 300 and ‘Nocturne’ x US 1212

In general, the differential expression prediction and RT-qPCR results agreed well with each other (Fig. 5a) and fit a linear regression model with coefficient of 0.62 (Pearson’s correlation, t = 4.27, df = 29, *p*-value = 1.91e-04). Two of the genes among the validated differentially expressed genes appeared to be possibly related to wax biosynthesis; one had homology to acyl-[acyl-carrier-protein] hydrolase, and the other had homology to HXXXD-type acyl-transferase. From the RT-qPCR results, the gene with homology to acyl-[acyl-carrier-protein] hydrolase was expressed at a 11.68 fold higher and 5.07 fold higher level, on average, in the individual plants that comprised the waxy bulks than the individual plants that comprised the non-waxy bulks of the ‘Nocturne’ x T 300 and ‘Nocturne’ x US 1212 populations, respectively (Fig. 5b). The gene with homology to HXXXD-type acyl-transferase was expressed at a 0.36 fold lower level in the waxy bulk plants of the ‘Nocturne’ x T 300 population and a 2.26 fold higher level in the waxy bulk plants of the ‘Nocturne’ x US 1212 population, making it a less likely candidate for the waxy gene in our populations (Additional file 4: Table S9). In Fig. 6, we show the levels of the acyl-[acyl-carrier-protein] hydrolase mRNA (from RT-qPCR) in fruit tissue of five individual plants of the waxy bulks and five individual plants of the non-waxy bulks from both populations. Of these 20 plants, all of the waxy plants had higher expression of the *FatB* gene than did the non-waxy plants. There was a range of expression levels among all individual plants tested (38 total; 10 waxy and 7 non-waxy from the ‘Nocturne’ x T 300 population, 13 waxy and 8 non-waxy from the ‘Nocturne’ x US 1212 population), and they are shown in the box plots displayed in Additional file 1: Figure S4.

Next we selected 17 of the major genes known to be involved in wax accumulation from the literature [6], all of which were included in our waxybase, for RT-qPCR analyses. These were chosen regardless of whether or not they were predicted to be differentially expressed based on the RNA-seq data. The RT-qPCR results indicated that none of these genes were significantly differentially expressed between the waxy and non-waxy bulks of either population (Additional file 5: Table S10).

### Sequence analysis of blueberry *FatB* cDNA and gDNA

**Fig. 7:**
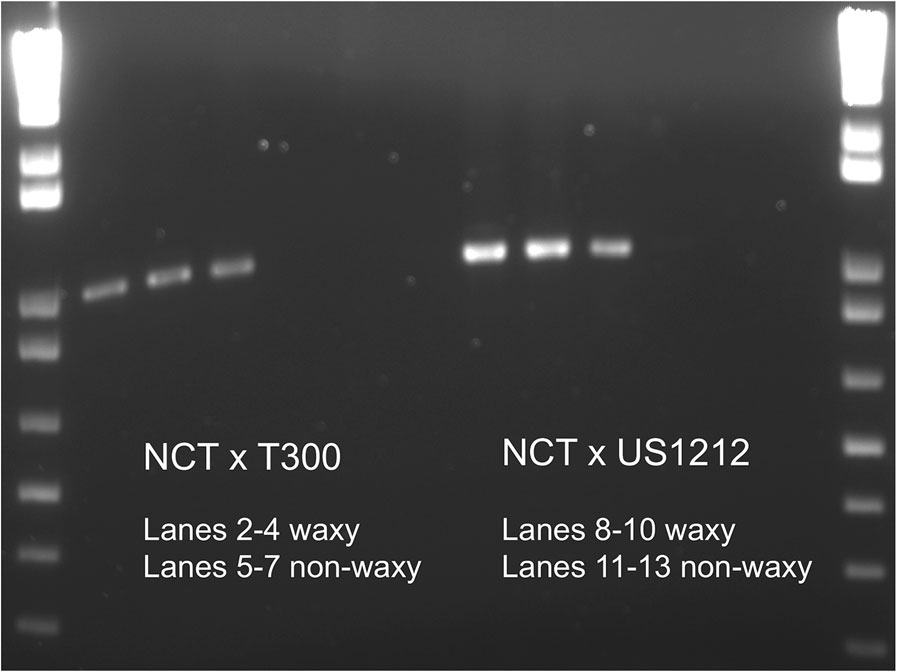
Attempts to amplify the cDNA for the differentially expressed acyl-[acyl-carrier-protein] hydrolase homolog, or *FatB* gene, from three of the waxy plants and three of the non-waxy plants of each of the two populations, ‘Nocturne’ x T 300 and ‘Nocturne’ x US 1212. Primers were designed near the ends of the assembled transcript sequence. Lanes 1 and 14: 1 kb plus ladder (MW standards, Invitrogen, Carlsbad, CA)

**Fig. 8:**
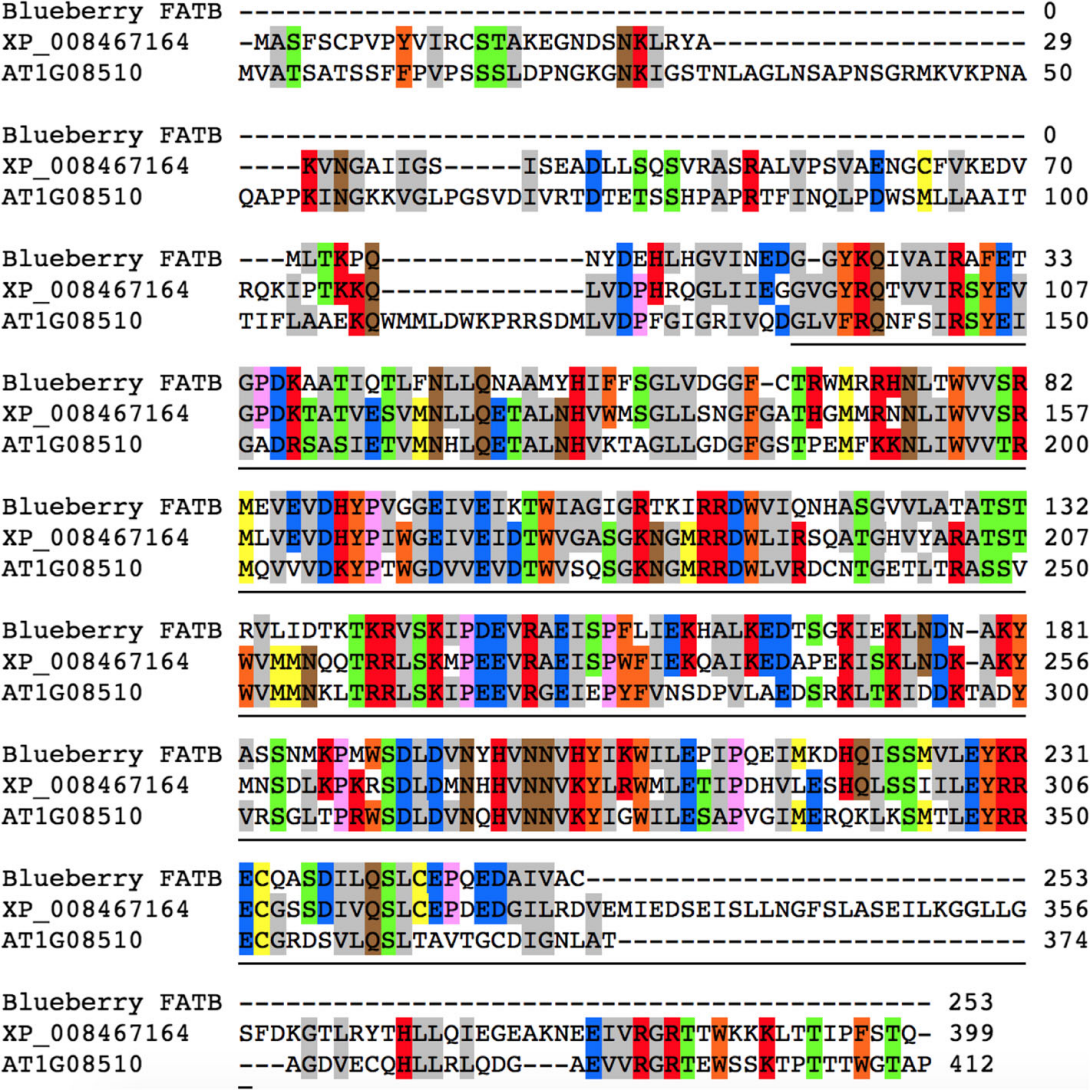
Alignment of deduced blueberry FATB protein, *Arabidopsis* FATB protein AT1G08510, and XP_008467164. XP_008467164 is the best hit from an NCBI BASTP search using the blueberry sequence. XP_008467164 is annotated as the *Cucumis melo* palmitoyl-acyl carrier protein thioesterase by NCBI

We attempted to amplify the cDNA for the differentially expressed acyl-[acyl-carrier-protein] hydrolase homolog, also known as FATB, from three of the waxy plants (with the highest level of expression of this gene) and three of the non-waxy plants (with the lowest level of expression) from each of the two populations by designing primers near the ends of the assembled transcript sequence. Amplification was successful for only the waxy plants, presumably because expression of the gene was too low in the non-waxy plants (Fig. 7). The cDNA amplification products from the six waxy plants were directly sequenced, without cloning first, and compared to each other. No differences were found in their sequences. We then conducted multiple sequence alignments between the *Vaccinium* deduced FATB protein sequence (253 amino acids), the *Cucumis melo* FATB (XP_008467164; annotated as palmitoyl-acyl carrier protein thioesterase, the best hit from an NCBI BLASTP search) protein sequence (Fig. 8), and FATB protein sequences from several other species (Additional file 1: Figure S5). The PF01643 domain (acyl-[acyl-carrier-protein] thioesterases, Acy-ACP-TE domain), shown by underlining in Fig. 8, gives this protein its catalytic function of terminating fatty acyl group extension by hydrolyzing the acyl group from the fatty acid. Within this domain, 80.08% of the amino acids were identical or had conservative replacements between the blueberry and the *Cucumis melo* sequences.

Because we could not amplify the cDNA sequence from the non-waxy plants that we tested, we also attempted to sequence the *FatB* gene itself. We designed primers according to the blueberry genome and sequenced the amplified genomic DNA fragments of both waxy and non-waxy plants of the two populations. We captured all exon regions of this gene, but did not sequence all the way through two very large introns. After aligning cDNA sequences back to the gDNA sequences, we determined that the blueberry *FatB* gene consists of six exons and five introns (Additional file 1: Figure S6). We compared the *FatB* sequence from waxy plants and non-waxy plants in both populations, and found only some degenerate nucleotide variations.

## Discussion

For perennial shrubs like blueberry, breeding a new variety can take 9 to 20 years from the original cross [16]. Genomic-assisted breeding has proven to be effective and efficient in some major crops and should be especially useful in perennial fruit trees and shrubs with their long generation times. Genomic resources are becoming available in blueberry. Genetic linkage maps of diploid [17] and commercial tetraploid [18] blueberry have been constructed but need further saturation. The blueberry genome has been estimated to be ~ 600 Mbp by flow cytometry [19]. A diploid blueberry (*V. corymbosum*) accession ‘W85–20’ has been sequenced and assembled to a length of 358 Mbp (15,129 scaffolds) using Roche’s 454 Newbler assembler [20] and annotated using Illumina RNA-seq combined with 454 sequence data, resulting in ~ 60,000 blueberry gene models [21]. Several blueberry transcriptome studies have been published focusing on revealing the underlying mechanisms of cold acclimation [10, 22, 23], fruit ripening [21, 24], and fruit antioxidant content [25]. To date, however, there is still no chromosome-level blueberry genome with dedicated gene annotation publicly available. In the present study, we report a collection of 91,861 blueberry unigenes assembled using our own RNA-seq data from this experiment in addition to RNA-seq data from other studies [21, 25]. This unigene set will provide high quality evidence for blueberry genome sequence annotation.

According to a recent survey reporting blueberry breeding trait priorities, firmness, shelf life and appearance are among the most important fruit quality traits to the industry [16]. The waxy coating gives blueberry fruit the appealing light blue “bloom”. It has also been recently reported that various components of cuticular wax are highly correlated (some positively and some negatively) with fruit weight loss and softening in storage [26]. In our study, we used two northern-adapted rabbiteye hybrid breeding populations that share the common parent ‘Nocturne’, which is a hexaploid black-fruited cultivar with no visible waxy coating on the berries. The populations segregate for the waxy coating on the fruit, which gives the fruit a light blue dusty color as opposed to the black color. By combining the concept of bulked segregant analysis and RNA-seq, we compared gene expression profiles in waxy and non-waxy bulks from the two populations and identified differentially expressed genes (DEGs) with at least a two-fold difference in expression level. Expression of the best candidate genes for the waxy coating from the RNA-seq analysis was then validated by RT-qPCR.

**Fig. 9:**
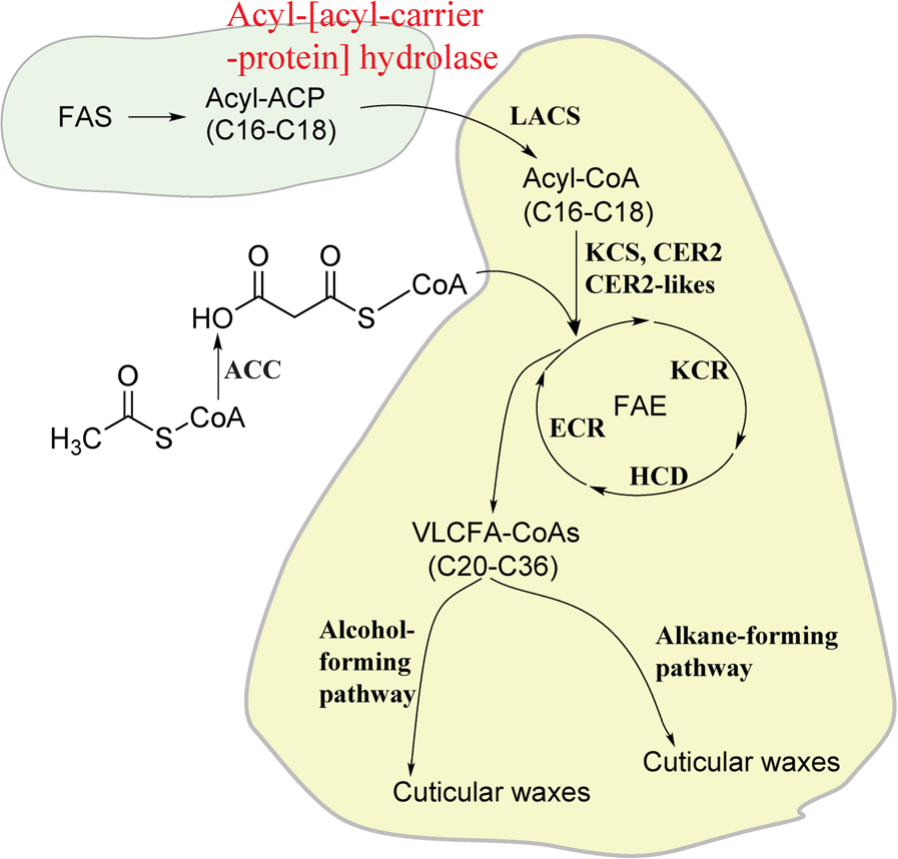
Diagram of cuticular wax biosynthesis pathway as taken from references [6, 30] showing Acyl-[acyl-carrier-protein] hydrolase location. Abbreviations: ACC: Acetyl-CoA carboxylase; CER: eceriferum; ECR: Enoyl-CoA reductase; FAE: Fatty acid elongase; HCD: beta-hydroxyacyl-CoA dehydratase; KCR: beta-ketoacyl-CoA reductase; KCS: beta-ketoacyl-CoA synthase; LACS: long-chain acyl-CoA synthetase

From this work, an excellent candidate gene emerged from the list of DEGs from the RNA-seq analysis, and its differential expression was validated by RT-qPCR on the individual plants which comprised the bulks, with an expression level that was 11.68 and 5.07-fold higher on average in the waxy bulks than in the non-waxy bulks of both populations. It was the only DEG that appeared related to wax biosynthesis and was expressed at more than a log_2_ two-fold higher level in the waxy bulks than the non-waxy bulks of both populations. The gene is a blueberry homolog to acyl-[acyl-carrier-protein] hydrolase, also called the *FatB* gene in *Arabidopsis*. In maize, insertions in the *FatB* gene (GRMZM5G829544) have been shown to result in reduced palmitic acid (16:0) content in the seeds [27, 28]. In *Arabidopsis*, a T-DNA insertion in the *FatB* gene (AT1G08510) has been shown to result in lower levels of palmitate (16:0) and stearate (18:0) in various tissue types, resulting in a reduction in growth rate. Furthermore, the *FatB* gene has been implicated in supplying fatty acids for wax biosynthesis, as the T-DNA insertion/knock-out mutation in *Arabidopsis* resulted in a reduction of 20 and 50% of the total wax load in leaves and stems, respectively, of the mutant [29]. This mutation causes a severe reduction in the supply of fatty acids for very-long-chain fatty acid (VLCFA) biosynthesis. Figure 9 shows how the products from the acyl-[acyl-carrier-protein] hydrolase reaction funnel into the VLCFA biosynthesis pathway, which in turn is required for wax biosynthesis. VLCFAs are modified by the alcohol-forming pathway or the alkane-forming pathway to produce the main components of cuticular wax [30].

We amplified and sequenced the cDNA for the blueberry *FatB* gene from three waxy plants of each population, but were unable to amplify the cDNA from the three non-waxy plants that were tested, presumably because of its low expression in these plants. We then amplified and sequenced a large portion of the *FatB* gene itself from waxy and non-waxy individuals of both populations. Alignment of the cDNA and gDNA sequences revealed that the blueberry *FatB* gene consists of six exons and five introns. Although we did not sequence through two very large introns, a comparison of the exon sequences found no significant sequence differences between the waxy and non-waxy plants, suggesting that another gene, which regulates or somehow affects *FatB* expression, must be segregating in the populations.

The segregation of the waxy coating on the fruit in our populations suggests that the trait is quantitative, as the waxy plants appear to have different levels of wax. Thus, the presence of the waxy coating should be determined by at least a few genes, although one gene could be responsible for a majority of the genotypic variance. The results from our study indicate that expression of the blueberry *FatB* gene is closely related to the waxy bloom on the fruit. However, our efforts to sequence the gene in waxy and non-waxy plants did not result in the identification of a mutation in this gene linked to the non-waxy phenotype. If a regulatory gene could be identified that controls *FatB* expression, and if it proves responsible for segregation of the fruit wax in this and other blueberry breeding populations, it could be developed for use as a marker in marker-assisted selection.

This study shows that expression of the *FatB* gene is related to the waxy phenotype, and the relationship between this gene and the waxy coating can be tested in other mapping and breeding populations of blueberry and related *Vaccinium* species, like cranberry and lingonberry. We are currently mapping the fruit color trait (from black to light blue) in our diploid blueberry mapping population and hope to determine if this gene or another wax-related gene coincides with a major QTL for the trait in the diploid population. Also, this study has resulted in an assembled transcriptome for blueberry, which provides high quality gene expression evidence for functional annotation of the emerging blueberry genome.

## Conclusions

In this study, we took advantage of two northern-adapted rabbiteye hybrid breeding populations that were segregating for the waxy coating on the fruit. We performed bulked segregant analysis combined with RNA-seq analysis. We assembled a set of 91,861 blueberry unigenes, which should help to provide expression evidence for blueberry genome sequence annotation. Using this assembly, we explored DEGs related to the waxy coating on fruit and identified a gene, *FatB*, whose expression is closely linked to presence of the epicuticular waxy layer.

## Methods

### Plant material

The two northern rabbiteye breeding populations used for this study resulted from the crosses ‘Nocturne’ [31] x T 300 (33 plants) and ‘Nocturne’ x US 1212 (36 plants). ‘Nocturne’ and US 1212 are complex mixed-species hexaploids composed primarily of *V. virgatum* and *V. constablaei* with lesser contributions of *V. corymbosum*, *V. darrowii*, and *V. tenellum*. ‘Nocturne’ and US 1212 originated from the USDA-ARS breeding program at Chatsworth, New Jersey. T 300 is 100% *V. virgatum*. T 300 originated from a cooperative breeding project between the USDA-ARS and the University of Georgia. ‘Nocturne’ is dark-fruited with little or no wax. Both populations were clearly segregating for presence or absence of a waxy coating on the fruit. In the summer of 2014, plants were given a rating from 0 to 5 based on the amount of wax which was visually apparent on the fruit. Scores of 0 or 1 indicated no or little wax, scores of 2 or 3 indicated presence of a medium amount of wax, and scores of 4 or 5 indicated presence of a heavy waxy coating. For preparation of the bulks (discussed below), only plants with a score of 0 were used in the non-waxy bulks; plants with a score of > 2 were used in the waxy bulks. Ripe fruit from each plant was flash frozen in liquid nitrogen and stored at − 80 °C for future RNA extractions (described below). The segregation ratios for this trait in both populations are described in Table 1.

### RNA extractions, cDNA synthesis and quality testing

For RNA-seq libraries, RNA was extracted from bulked tissue samples. The bulks of ‘Nocturne’ x T 300 consisted of 10 waxy individuals and 9 non-waxy individuals, and for the ‘Nocturne’ x US 1212 population, bulks were comprised of 13 waxy individuals and 10 non-waxy individuals. To make each bulk, an equal amount of fruit tissue (0.5 g) from each individual of each type was used. RNA was then extracted as previously described [10]. Following extractions, RNA concentration and quality were measured on a NanoDrop ND-1000 (NanoDrop Technologies, Wilmington, DE, USA). Additionally, quality was checked on a 1% agarose gel stained with ethidium bromide.

For real-time PCRs, RNA was extracted from individuals that had comprised the waxy and non-waxy bulks of both populations. Four grams of fruit tissue were used, and the RNA was extracted using the same procedure used for the bulks [10]. RNA concentration and purity were measured on a NanoDrop ND-1000. Only the RNA samples with A260/A280 ratios between 1.9 and 2.1 and A260/A230 greater than 2.0 were used in further analyses. To verify integrity, we amplified two 101 bp long cDNA segments of the 5′ and 3′ regions of an ubiquitin carboxyl-terminal hydrolase gene (*UBP14*) across the cDNA samples by qPCR. The fragments are 1769 and 348 bp, respectively, from the 3′ end of the cDNA. The 3′:5′ amplification ratios of the *UBP14* cDNA fragments were calculated from all samples using the comparative Cq method [32]. All ratios fell within the range of 1.28–3.06 (2.44 ± 0.89; mean ± SD). Only if ratios were > 4.4 would RNA quality be deemed inadequate [33]. To remove contaminating genomic DNA (gDNA), before cDNA synthesis, RNA extracts were treated with TURBO™ DNase I (Life Technologies, USA), as previously described [22]. Following cDNA synthesis, cDNA samples were tested for gDNA contamination also as described previously [22]. In tests for gDNA contamination, the 1140 bp band was not amplified from any of the samples. Because the cDNA samples met our criteria for RNA quality and were gDNA contamination-free, they were judged to be suitable for qPCR analysis.

### Sequencing and data trimming

Two RNA-seq libraries from the waxy and non-waxy bulks of each of the two blueberry populations described above were constructed following Illumina HiSeq2500 manufacturer’s instructions. Libraries were prepared and sequenced at the David H. Murdock Research Institute in Kannapolis, NC. Paired-end reads of 100 bp were generated. Raw reads were trimmed based on two criteria. First, 10 nucleotides from the 5′ ends and 5 nucleotides from the 3′ ends were discarded to remove residue adaptor sequences and low quality sequences from the ends. After this, more than 94% of the remaining base pairs had a per base quality score > 30. Second, any reads containing more than 10 ambiguous nucleotides (out of 85 bp) were discarded.

All available blueberry RNA-seq data published before 2016 were retrieved from the National Center for Biotechnology Information (NCBI) Short Read Archive (SRA), including libraries under accession numbers SRP039977, SRP039971 and SRA046311. Downloaded data were subjected to quality trimming using the same criteria described above. Read quality score statistics were given by FastQC [34].

### De novo assembly and reference-based assembly

De novo blueberry transcriptome assembly was performed using all RNA-seq data generated from this study and RNA-seq data downloaded from the SRA. Laddered assembly was performed based on different millions of randomly selected reads from all the data. All assemblies were carried out using Trinity [35] (version 2.1.1) with parameters set as follows: --KMER_SIZE = 25, −-normalize_reads, −-normalize_max_read_cov = 60.

In addition, a reference-guided assembly was performed using a TopHat-Cufflinks protocol [36]. TopHat was used to align all RNA-seq data to the blueberry reference genome and generate BAM files. All BAM files were sorted using SAMtools [37]. Sorted BAM files were then utilized by Cufflinks for transcript assembly. For each of the libraries, Cufflink generated a GTF file. CUFFMERGE was then used to merge all GTF files into a single GTF file.

Two different methods were performed to assess the quality of each assembly. First, RNA-seq read representation was evaluated by mapping clean reads back to the reference genome using TopHat [38], and mapping clean reads back to the assemblies, both the reference-based and de novo versions, using Bowtie [39]. Second, assembled transcripts were examined for the number of full-length hits by BLASTN to the draft blueberry genome annotation (Robert Reid, UNC, and Allan Brown, IITA, personal communication) and BLASTP to SwissProt/Trembl [12] recorded proteins. The best hits from all alignments were selected from both BLASTN and BLASTP searching results.

Reference-based assembled transcripts were also aligned against de novo assembled transcripts using BLASTN. Resulting hits of alignment with identity scores > 80, query lengths > 80%, and subject lengths > 80 were marked as high confidence transcripts

### Unigene assignment and functional annotation

Coding regions within assembled transcripts were further identified using TransDecoder [40]. ORFs were predicted and mapped back to SwissProt protein database using BLASTP and mapped back to Pfam domain database using HMMscan [41].

To eliminate redundancy within the assemblies, the transcripts with the longest predicted CDS were selected for each gene, and transcripts with a predicted CDS < 200 bases were discarded. In addition, CD-HIT [13] with default parameters (version 4.7, built on May 1, 2017) was used to eliminate redundant transcripts. Finally, TIGR Gene Indices CLustering tools (TGICL) [14] was used to double check for redundancy (minimum overlap length = 120, minimum percent identity for overlaps = 90).

Protein records from NCBI Non-redundant protein sequence database (Nr), SwissProt/Trembl, PlantCyc [42] and NCBI RefSeq [43] release plant (refPlant) were downloaded to our local server and formatted into protein databases. For functional annotation, BLASTP was performed on unigene deduced protein sequences against each database. The resulting hits were filtered by the criteria: identity > = 40, hit score > =60, and hit length > = half of the query sequence length. The BlastKOALA [44] web server was used for KEGG sub-database “genus_eukaryotes.pep” annotation.

### Identification of differentially expressed transcripts

For alignment-based abundance evaluation, clean reads from each of the four different RNA-seq libraries (from waxy and non-waxy bulks from the two populations) were mapped back to our blueberry unigene dataset using Bowtie. Expression abundance was then evaluated using the RNA-Seq by Expectation Maximization (RSEM) method [45] for each library. Differential expression level was identified using edgeR package [15]. For alignment-free expression quantification, Salmon [46] was performed based on a two-phase interference procedure.

### Waxybase construction

A list of genes related to wax accumulation in plants was generated based on the literature. Protein sequences of such genes were retrieved from Phytozome [47] or from NCBI. Sequences from the plant cuticular wax biosynthesis pathway PWY-282 were downloaded directly from PlantCyc [42]. BLASTP was used to identify the best matches between our wax database (waxybase) and the blueberry unigene dataset. Filtering criteria included: identity > 70, > 70% length of unigene protein sequence aligned, and alignment score > 100. For those genes where we could not find good homology with blueberry sequences, we searched for grape homologs instead.

### Real-time qPCR primer design

The NCBI BLAST software was used to test the specificity of all PCR primers. Primers for real-time qPCR were designed utilizing the criteria: Tm of 60 ± 2 °C, PCR amplicon lengths of 65–100 bp, primer sequences of 20–23 nucleotides in length, and GC contents of 40–60%. The secondary structure of the amplicons was predicted from MFOLD version 3.4 software (default settings of minimal free energy, 50 mM Na^+^, 3 mM Mg^2+^, annealing temperature of 60 °C) [48]. Primers were chosen that gave amplicons with minimal secondary structures as well as melting temperatures that would not interfere with annealing. Integrated DNA Technologies (Coralville, IA, USA) synthesized the primers.

### Real-time qPCR experiment

PCR reactions were carried out in an IQ5 (Bio-Rad, Hercules, CA, USA) thermal cycler using iQ™ SYBR® Green Supermix. Reactions contained 1 μl of diluted cDNA as a template and 0.150 μM of each primer in a total volume reaction of 20 μl. The following thermal profile was used for all PCRs: polymerase activation (95 °C for 3 min), amplification and quantification cycles repeated 40 times (95 °C for 30 s, 60 °C for 1 min). The specificity of the primer pairs was checked by melting-curve analysis and amplification plots were analyzed as previously described [22]. Normalized relative quantities (NRQ) were determined as described in Hellemans et al [49]. The overall mean efficiency of real-time PCR amplification for each primer pair (E) was determined from the exponential phase of individual amplification plots. To calculate the efficiency, the eq. (1 + E) =10^slope^ was used with LinReg software. Three-five fluorescent data points with R^2^ ≥ 0.998 defined the linear regression lines [50, 51]. Normalization was performed using two stably expressed blueberry reference genes identified previously: *UBC28* and *Vc4g26410* [51] . To confirm the stable expression of the references in the current material, we calculated the mean of their NRQ (mean ± sd) for each waxy and non-waxy group of the two populations: (1) ‘Nocturne’ x T 300 population, *UBC28* waxy plants = 1.16 ± 0.29, non-waxy = 1.14 ± 0.15, *Vc4g26410* waxy plants = 0.91 ± 0.22, non-waxy = 0.89 ± 0.10; (2) ‘Nocturne’ x US 1212 population, *UBC28* waxy plants = 1.19 ± 0.18, non-waxy = 1.30 ± 0.23, *Vc4g26410* waxy plants = 0.86 ± 0.14, non-waxy = 0.79 ± 0.14.

### Sequencing cDNA and genomic DNA of best candidate gene(s)

Attempts were made to amplify a near full-length cDNA of a gene whose expression was related to the waxy coating (the *FatB* gene which encodes acyl-[acyl-carrier-protein] hydrolase), from three waxy and three non-waxy plants of each population that were included in the original bulks. Complementary DNAs were synthesized by priming with oligo-dT12–18 (Life Technologies, USA), using SuperScriptIII reverse transcriptase following the instructions of the provider. The cDNAs were diluted to a final volume of 50 μl. PCR primers were designed based on the assembled transcript sequence and genome annotation information [20] (Forward primer CATGCTTTCACGTTGCAGAT; Reverse primer CCGTCTCTCCTTGGATTTGA). PCR reaction volumes were 20 μL containing 1x Promega (Madison, WI) GoTaq Flexi Buffer, 3 mM MgCl_2_, 0.2 mM each dNTP, 0.1 μM each of the forward and reverse primers, 0.5 units Promega GoTaq Flexi DNA polymerase and 1 μL cDNA. Amplification was carried out in a Bio-Rad (Hercules, CA) T100 thermal cycler with the following profile: an initial denaturation of 95^°^ for 5 min, then 40 cycles of denaturation (92^°^, 40s), annealing (60^°^, 30s), extension (72^°^, 60s), and a final extension step at 72^°^ for 10 min.

To amplify the *FatB* gene for sequencing, total genomic DNA was first extracted as previously described [52] from 3 waxy and 3 non-waxy plants of each population that were included in the original bulks. PCR primers were designed based on gene models deduced by aligning FATB cDNA sequence to blueberry genome and cranberry genome. PCR reaction volumes were 20 μL containing 1x Promega (Madison, WI) GoTaq Flexi Buffer, 3 mM MgCl2, 0.2 mM each dNTP, 0.1 μM each of the forward and reverse primers, 0.5 units Promega GoTaq Flexi DNA polymerase and 25 ng genomic DNA. Amplification was carried out in a Bio-Rad (Hercules, CA) T100 thermal cycler with the following profile: an initial denaturation of 95° for 5 min, then 40 cycles of denaturation (92°, 40s), annealing (60°, 60s), extension (72°, 120 s), and a final extension step at 72° for 10 min.

Amplification products were purified using the Zymoclean Gel DNA Recovery Kit (Zymo Research, Irvine, CA), direct sequenced using the ABI Big Dye Terminator v3.1 Cycle Sequencing Kit (Applied Biosystems, Foster City, CA) according to the manufacturer’s protocol, and run on an Applied Biosystems 3500 Genetic Analyzer.

### Sequence assembly of *FatB* gene

Raw sequenced reads were quality trimmed and then anchored by the order of their primer template position referenced to the blueberry genome sequence. Overlaps among anchored reads were determined by BLASTN. Multiple sequence alignments were performed by ClustalW [53] to identify possible variations. The cDNA sequence was aligned back to the assembled gDNA sequence using BLASTN to determine possible exon/intron boundaries.

## Supplementary information

**Additional file 1.** Supplementary Information.

**Additional file 2: Table S7.** Predicted DEGs in ‘Nocturne’ x T 300 population.

**Additional file 3: Table S8.** Predicted DEGs in ‘Nocturne’ x US 1212 population.

**Additional file 4: Table S9.** Real-time qPCR data for selected DEGs. Fold change waxy /non-waxy is shown as mean of the normalized relative quantities ± SEM.

**Additional file 5: Table S10.** Real-time qPCR data for 17 major genes involved in wax accumulation selected from literature.

## Data Availability

All RNA-seq reads generated by this study are publicly available at the NCBI Short Read Archive (SRA) under accession numbers SRR6281886, SRR6281887, SRR6281888 and SRR6281889. The assembled transcriptome is deposited at DDBJ/EMBL/GenBank Transcriptome Shotgun Assembly (TSA) database under the accession number GGAB00000000. Complementary DNA sequence of blueberry FATB is deposited under accession number SRR7879249.

## Abbreviations

ABCG: half-transporters must dimerize to form functional ABC transporter
ACC: Acetyl-CoA carboxylase
ACLA2: ATP-citrate lyase subunit A2
BDG3: hydrolase BODYGUARD 3
CD1: tomato extracellular acyltransferase
CER: eceriferum
ECR: Enoyl-CoA reductase
FAE: Fatty acid elongase
FAR: Fatty acyl-CoA reductase
FATB: acyl-ACP thioesterases
FDH: FIDDLEHEAD
GL8: Glossy8 gene, beta-ketoacyl reductase
GPAT6: Glycerol-3-phosphate acyltransferase 6
HCD: beta-Hydroxyacyl-CoA dehydratase
HTH: HOTHEAD
KCR: beta-Ketoacyl-CoA reductase
KCS: beta-ketoacyl-CoA synthase
LACS: long-chain Acyl-CoA synthetase
LCR: encodes cytochrome P450 CYP86A8, fatty acid ω-hydroxylase
PAS2: very-long-chain hydroxy fatty acyl-CoA dehydratase
RDR1: RNA-dependent RNA polymerase 1
SlTTS1: monofunctional β-amyrin synthase
WIN1/SHN1: WAX INDUCER1, AP2-domain protein transcription factor
WXP1: AP2 domain-containing putative transcription factor
