# Supplementary material for: Transcriptome analysis identifies genes related to the waxy coating on blueberry fruit in two northern-adapted rabbiteye breeding populations
Source: BMC Plant Biol. 2019 Oct 31;19:460. doi: 10.1186/s12870-019-2073-7 (PMC6844065; doi:10.1186/s12870-019-2073-7)
Supplement: Supplementary file 1 — Additional file 1. Supplementary Information. [file 12870_2019_2073_MOESM1_ESM.docx]

**
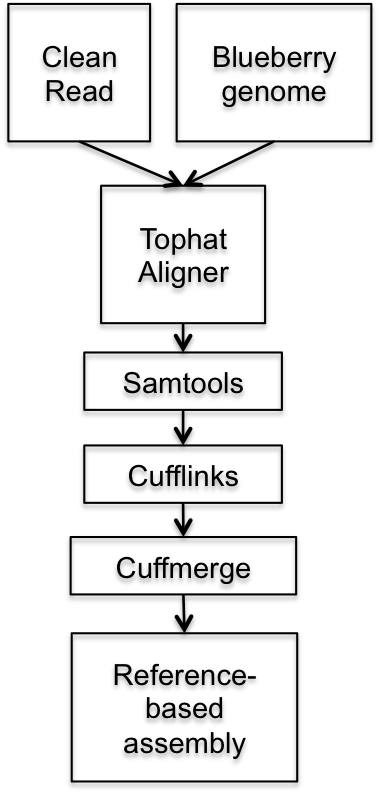
**

**Supplementary Figure 1.** Workflow of reference-based blueberry transcriptome assembly.


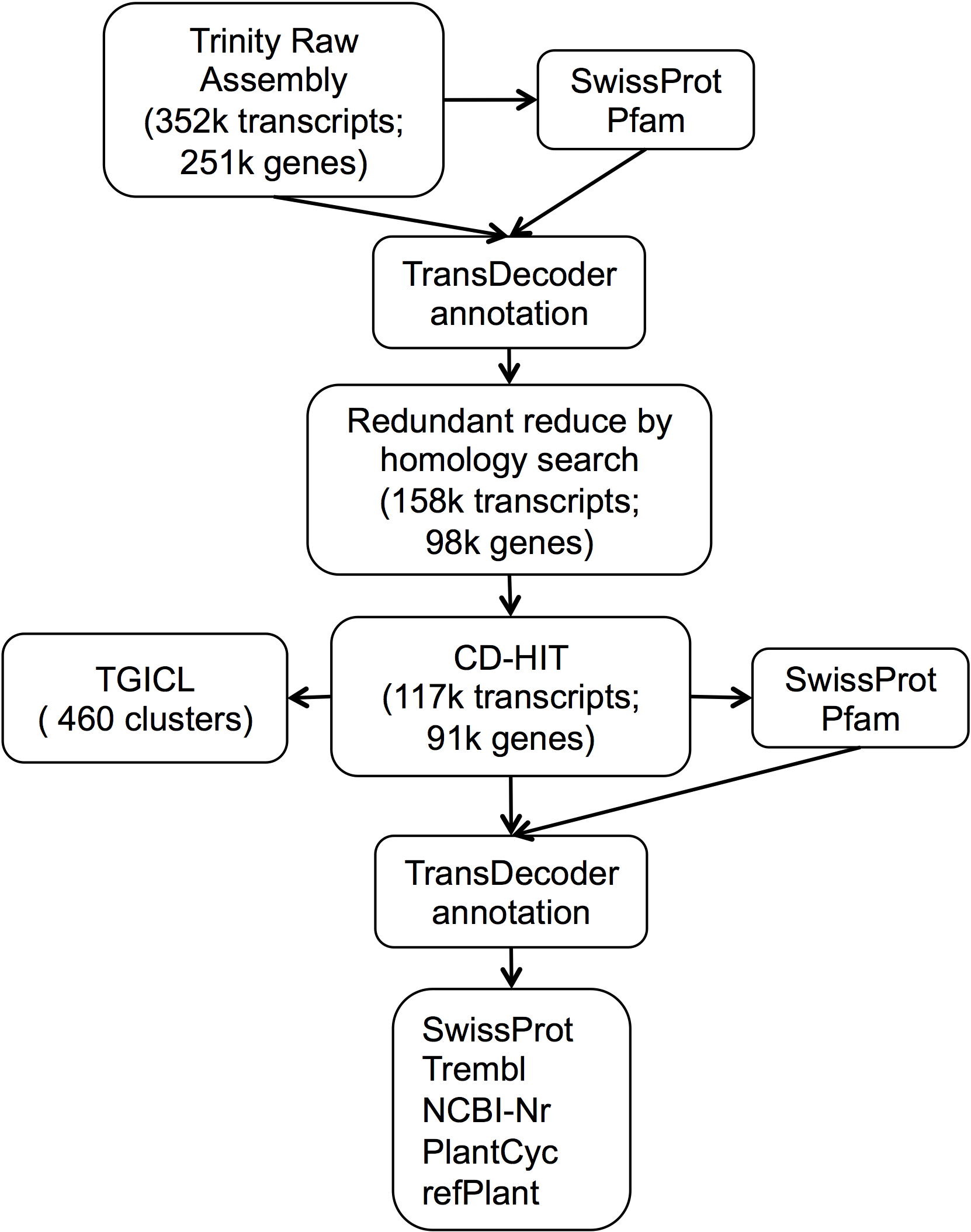


**Supplementary Figure 2.** Workflow of *de novo* transcriptome assembly, gene model filtration and gene function annotation.


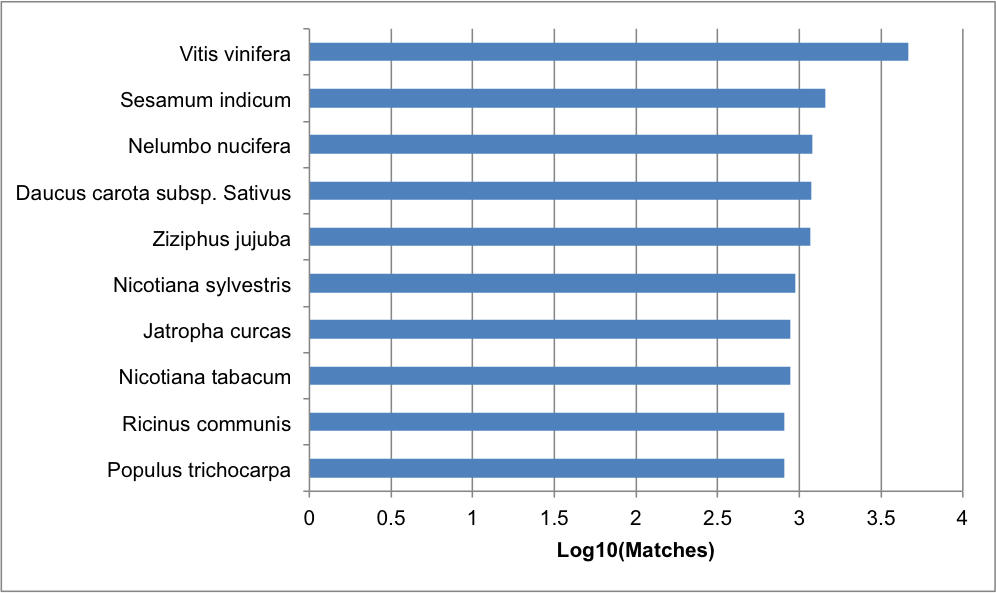


**Supplementary Figure 3.** Top 10 species in NCBI refPlant database with the most hits of blueberry annotated unigenes.


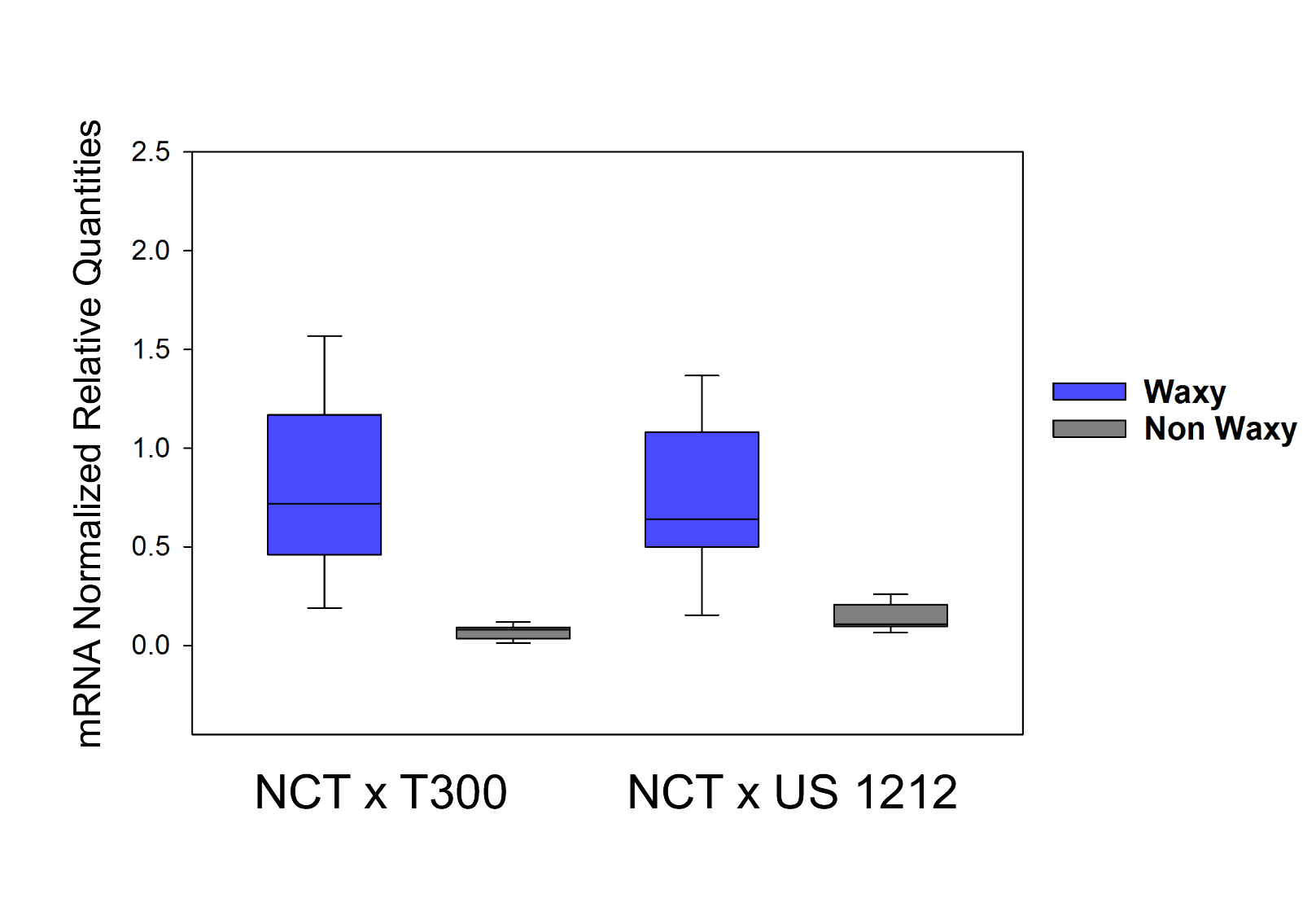


**Supplementary Figure 4.** Box plots showing the range of *FatB* expression levels (RT-qPCR results) in the waxy and non-waxy plants that were tested in each population (38 total plants; 10 waxy and 7 non-waxy from the ‘Nocturne’ x T 300 population, 13 waxy and 8 non-waxy from the ‘Nocturne’ x US 1212 population). Lines through the boxes indicate the median values. Mean values indicate that the gene was expressed at an 11.68 fold higher level and a 5.07 fold higher level on average in the waxy plants than in the non-waxy plants of the ‘Nocturne’ x T 300 population and the ‘Nocturne’ x US 1212 population, respectively.


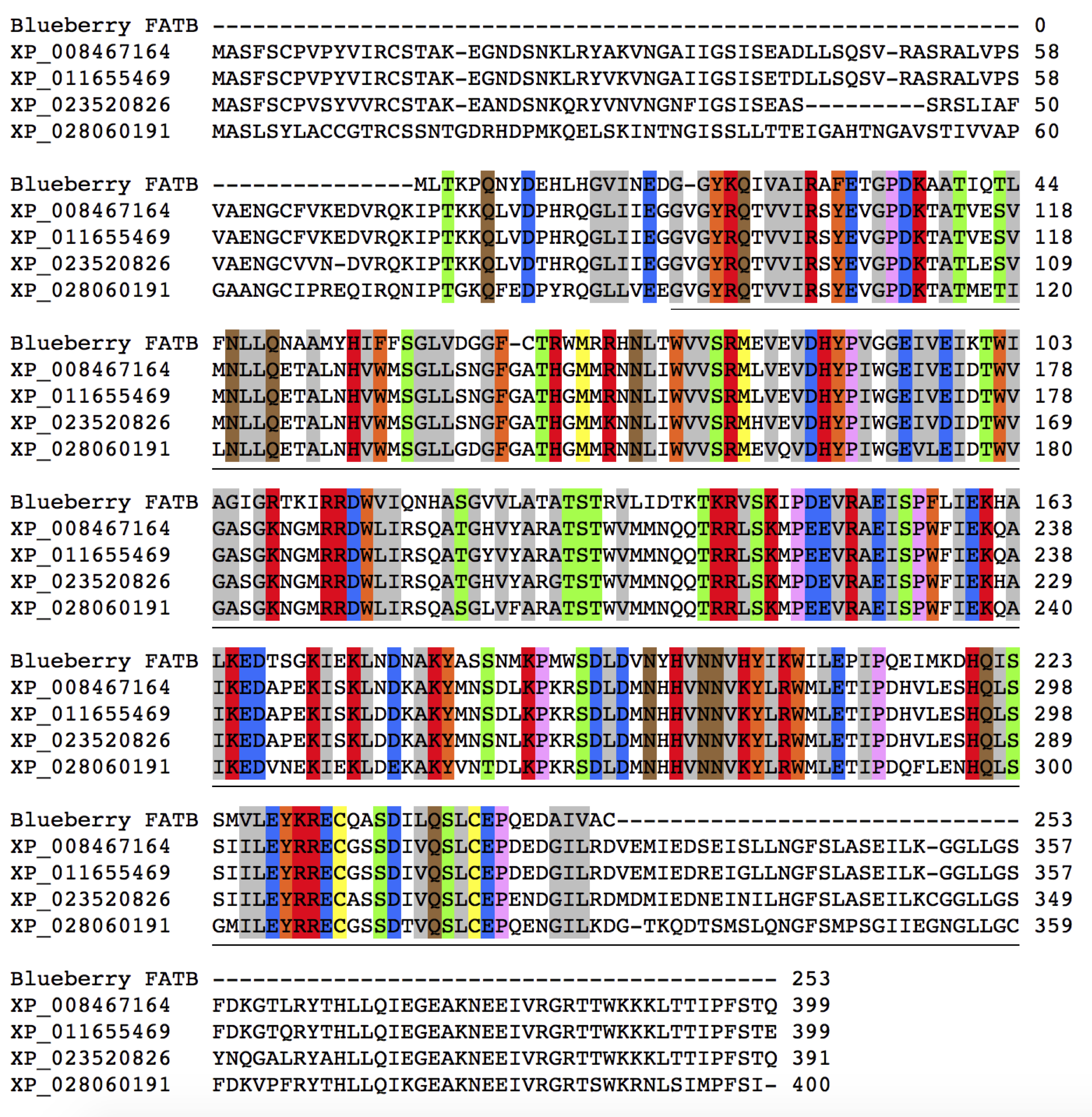


**Supplementary Figure 5.** Alignment of deduced amino acid sequence of blueberry FATB protein and FATB protein of other plant species. XP_008467164, XP_011655469, XP_023520826, and XP_028060191 are from species *Cucumis melo, Cucumis sativus, Cucurbita pepo subsp. pepo*, and *Camellia sinensis* respectively.





**Supplementary Figure 6.** A. Alignment of blueberry *FatB* genomic DNA sequences against blueberry reference genome. Blue color was used for waxy sequences and black color was used for non-waxy sequences. **B**. Deduced gene structure of blueberry *FatB* gene. Exon-intron boundaries were determined by aligning FATB-cDNA sequence against assembled *FatB* genomic DNA sequence.

**Supplementary Table 1.** Summary of RNA-seq reads from two northern-adapted rabbiteye hybrid blueberry breeding populations.

| **Library ID** | **Raw Read**  **Number** | **Clean Read**  **Number** | **Length**  **(bp)** | **Total (bp)** |
| --- | --- | --- | --- | --- |
| NCTxT300NonWaxy_R1 | 34,689,099 | 34,659,111 | 85 | 2,946,024,435 |
| NCTxT300NonWaxy_R2 | 34,689,099 | 34,659,111 | 85 | 2,946,024,435 |
| NCTxT300Waxy_R1 | 48,857,578 | 48,814,894 | 85 | 4,149,265,990 |
| NCTxT300Waxy_R2 | 48,857,578 | 48,814,894 | 85 | 4,149,265,990 |
| NCTxUS1212NonWaxy_R1 | 34,140,928 | 34,111,279 | 85 | 2,899,458,715 |
| NCTxUS1212NonWaxy_R2 | 34,140,928 | 34,111,279 | 85 | 2,899,458,715 |
| NCTxUS1212Waxy_R1 | 40,737,484 | 40,702,210 | 85 | 3,459,687,850 |
| NCTxUS1212Waxy_R2 | 40,737,484 | 40,702,210 | 85 | 3,459,687,850 |

**Supplementary Table 2.** Blueberry RNA-seq reads mapping results.

| **Sample** | **Genome (%)** | **Reference-based Assembly (%)** | ***De novo* Assembly (%)** |
| --- | --- | --- | --- |
| NCTxT300NonWaxy | 72.5 | 65.6 | 87.2 |
| NCTxT300Waxy | 82.3 | 73.6 | 92.0 |
| NCTxUS1212NonWaxy | 82.2 | 73.1 | 93.4 |
| NCTxUS1212Waxy | 82.1 | 73.5 | 91.6 |
| SRR942391 | 70.8 | 70.8 | 89.4 |
| SRR950441 | 72.7 | 70.3 | 89.7 |
| SRR1187632 | 61.5 | 57.3 | 71.5 |
| SRR1187673 | 65.4 | 60.9 | 78.6 |
| SRR1187674 | 1.4 | 1.2 | 2.9 |
| SRR1187675 | 56.7 | 52.8 | 67.2 |
| SRR1187676 | 61.0 | 56.8 | 74.1 |
| SRR1187677 | 63.4 | 59.5 | 75.0 |
| SRR1188088 | 53.0 | 57.1 | 73.6 |
| SRR1188089 | 55.3 | 59.4 | 73.3 |
| SRR1188090 | 54.7 | 59.2 | 74.7 |
| SRR1188091 | 55.2 | 59.2 | 72.8 |
| SRR1188222 | 56.1 | 59.7 | 73.4 |
| SRR1188230 | 60.1 | 59.7 | 75.5 |
| SRR1188236 | 60.3 | 59.8 | 74.4 |
| SRR1188240 | 60.1 | 59.9 | 75.7 |
| SRR1188242 | 60.5 | 59.8 | 73.8 |
| SRR1188247 | 57.3 | 58.5 | 74.1 |
| SRR1188258 | 61.4 | 60.0 | 73.9 |
| SRR1188265 | 58.0 | 58.7 | 72.4 |
| SRR1188270 | 55.4 | 56.2 | 70.0 |
| SRR1188282 | 57.3 | 58.6 | 74.2 |
| SRR1188283 | 53.2 | 54.6 | 70.2 |

**Supplementary Table 3.** Comparison between 454 assembled contigs and *de novo* assembly.

| Libraries | Number of hit | Number of contig | Percentage | Average identity |
| --- | --- | --- | --- | --- |
| Berries | 6,002 | 6,726 | 89.24 | 87.91 |
| Bud | 9,176 | 10,350 | 88.66 | 87.29 |
| MID10 | 2,305 | 2,675 | 86.17 | 84.67 |
| MID1 | 2,282 | 2,645 | 86.28 | 84.94 |
| MID2 | 2,071 | 2,421 | 85.54 | 84.14 |
| MID3 | 1,698 | 1,941 | 87.48 | 86.08 |
| MID4 | 2,354 | 2,751 | 85.57 | 84.21 |
| MID5 | 1,510 | 1,781 | 84.78 | 83.39 |
| MID6 | 1,942 | 2,241 | 86.66 | 85.32 |
| MID7 | 1,744 | 2,029 | 85.95 | 84.61 |
| MID8 | 1,710 | 1,964 | 87.07 | 85.66 |

Notes: BlastN results of hit length >= 75% of the 454 contig sequence length and results of hit length >= 500 bp were taken into account in this statistic.

**Supplementary Table 4.** Summary of raw *de novo* assembly statistic.

| Total trinity 'genes': | 251,974 |
| --- | --- |
| Total trinity transcripts: | 352,293 |
| Percent GC | 43 |
| Stats based on ALL transcript contigs: |  |
| Contig N10 | 2,702 |
| Contig N20 | 1,935 |
| Contig N30 | 1,458 |
| Contig N40 | 1,099 |
| Contig N50 | 806 |
| Median contig length | 361 |
| Average contig | 591 |
| Total assembled bases | 208,124,831 |
| Stats based on ONLY LONGEST ISOFORM per 'GENE': |  |
| Contig N10 | 2,503 |
| Contig N20 | 1,694 |
| Contig N30 | 1,184 |
| Contig N40 | 832 |
| Contig N50 | 610 |
| Median contig length | 327 |
| Average contig | 512 |
| Total assembled bases | 128,974,473 |

**Supplementary Table 5.** Published genes incorporated in waxybase.

| GeneID | Species | GeneName | FunctionClass | Reference |
| --- | --- | --- | --- | --- |
| AT1G01120.1 | *Arabidopsis thaliana* | KCS1 | Biosynthesis | ^1^ |
| AT1G01600.1 | *Arabidopsis thaliana* | CYP86A4 | Biosynthesis cutin | ^2^ |
| AT1G02205.2 | *Arabidopsis thaliana* | CER1 | Biosynthesis | ^3^ |
| AT1G02205.1 | *Arabidopsis thaliana* | CER1 | Biosynthesis | ^3^ |
| AT1G04220.1 | *Arabidopsis thaliana* | KCS2 | Biosynthesis | ^1^ |
| AT1G08510.1 | *Arabidopsis thaliana* | FATB | Biosynthesis | ^4^ |
| AT1G14790.1 | *Arabidopsis thaliana* | RDR1 | Regulation | ^5^ |
| AT1G15360.1 | *Arabidopsis thaliana* | WIN1/ SHN1 | Regulation cutin | ^6^ |
| AT1G17840.1 | *Arabidopsis thaliana* | ABCG11/WBC11 | Secretion | ^7^ |
| AT1G27950.1 | *Arabidopsis thaliana* | LTPG1 | Secretion | ^8^ |
| AT1G49340.1 | *Arabidopsis thaliana* | LACS2 | Biosynthesis | ^1^ |
| AT1G49340.2 | *Arabidopsis thaliana* | LACS2 | Biosynthesis | ^1^ |
| AT1G49430.1 | *Arabidopsis thaliana* | LACS2 | Biosynthesis | ^9^ |
| AT1G51460.1 | *Arabidopsis thaliana* | ABCG13 | Secretion | ^10^ |
| AT1G51500.1 | *Arabidopsis thaliana* | ABCG12/CER5 | Secretion | ^11^ |
| AT1G57750.2 | *Arabidopsis thaliana* | MAH1 |  | ^12^ |
| AT1G60810.1 | *Arabidopsis thaliana* | ACLA2 | Biosynthesis | ^1^ |
| AT1G64400.1 | *Arabidopsis thaliana* | LACS3 | Biosynthesis | ^13^ |
| AT1G64670.1 | *Arabidopsis thaliana* | BDG | Biosynthesis cutin | ^14^ |
| AT1G67730.1 | *Arabidopsis thaliana* | KCR1 | Biosynthesis | ^15^ |
| AT1G68530.1 | *Arabidopsis thaliana* | CER6 | Biosynthesis | ^16^ |
| AT1G68530.2 | *Arabidopsis thaliana* | CER6 | Biosynthesis | ^16^ |
| AT1G72970.1 | *Arabidopsis thaliana* | HTH | ENIGMATIC FACTORS | ^14^ |
| AT1G72970.2 | *Arabidopsis thaliana* | HTH | ENIGMATIC FACTORS | ^14^ |
| AT2G26250.1 | *Arabidopsis thaliana* | FDH | Biosynthesis | ^17^ |
| AT2G26910.1 | *Arabidopsis thaliana* | ABCG32 | Secretion | ^18^ |
| AT2G33510.2 | *Arabidopsis thaliana* | CFL1 | Regulation | ^19^ |
| AT2G33510.1 | *Arabidopsis thaliana* | CFL1 | Regulation | ^19^ |
| AT2G38110.1 | *Arabidopsis thaliana* | GPAT6 | Biosynthesis cutin | ^2^ |
| AT2G45970.1 | *Arabidopsis thaliana* | LCR | Biosynthesis cutin | ^20^ |
| AT2G47240.1 | *Arabidopsis thaliana* | LACS1/CER8 | Biosynthesis | ^9^ |
| AT2G47240.2 | *Arabidopsis thaliana* | LACS1/CER8 | Biosynthesis | ^9^ |
| AT3G01140.1 | *Arabidopsis thaliana* | MYB106 | Regulation | ^21^ |
| AT3G04290.1 | *Arabidopsis thaliana* | LTL1 | Biosynthesis cutin | ^6^ |
| AT3G10570.1 | *Arabidopsis thaliana* | CYP77A6 | Biosynthesis cutin | ^2^ |
| AT3G23840.1 | *Arabidopsis thaliana* | CER26-like | Biosynthesis | ^22^ |
| AT3G28910.1 | *Arabidopsis thaliana* | MYB30 | Regulation | ^23^ |
| AT3G43720.1 | *Arabidopsis thaliana* | LTPG2 | Secretion | ^8^ |
| AT3G43720.2 | *Arabidopsis thaliana* | LTPG2 | Secretion | ^8^ |
| AT3G47600.1 | *Arabidopsis thaliana* | MYB94 | Regulation | ^24^ |
| AT3G55360.1 | *Arabidopsis thaliana* | CER10 | Biosynthesis | ^25^ |
| AT3G56700.1 | *Arabidopsis thaliana* | FAR6 | Biosynthesis | ^26^ |
| AT3G60500.1 | *Arabidopsis thaliana* | CER7 | Regulation | ^27^ |
| AT3G60500.2 | *Arabidopsis thaliana* | CER7 | Regulation | ^27^ |
| AT3G60500.3 | *Arabidopsis thaliana* | CER7 | Regulation | ^27^ |
| AT3G61150.1 | *Arabidopsis thaliana* | HDG1 | Biosynthesis cutin | ^19^ |
| AT4G13840.1 | *Arabidopsis thaliana* | CER26 | Biosynthesis | ^22^ |
| AT4G22490.1 | *Arabidopsis thaliana* | LTP6 | Biosynthesis | ^1^ |
| AT4G23850.1 | *Arabidopsis thaliana* | LACS4 | Biosynthesis | ^28^ |
| AT4G24140.1 | *Arabidopsis thaliana* | BDG3 | Biosynthesis cutin | ^6^ |
| AT4G24510.1 | *Arabidopsis thaliana* | CER2 | Unknown | ^29^ |
| AT4G28110.1 | *Arabidopsis thaliana* | MYB41 | Regulation | ^30^ |
| AT4G34100.1 | *Arabidopsis thaliana* | CER9 | Biosynthesis | ^31^ |
| AT4G34100.2 | *Arabidopsis thaliana* | CER9 | Biosynthesis | ^31^ |
| AT5G10480.3 | *Arabidopsis thaliana* | PAS2 | Biosynthesis | ^32^ |
| AT5G10480.1 | *Arabidopsis thaliana* | PAS2 | Biosynthesis | ^32^ |
| AT5G10480.2 | *Arabidopsis thaliana* | PAS2 | Biosynthesis | ^32^ |
| AT5G11190.1 | *Arabidopsis thaliana* | SHN2 | Regulation cutin | ^6^ |
| AT5G15310.1 | *Arabidopsis thaliana* | MYB16 | Regulation | ^21^ |
| AT5G15310.2 | *Arabidopsis thaliana* | MYB16 | Regulation | ^21^ |
| AT5G23570.1 | *Arabidopsis thaliana* | SGS3 | Regulation | ^5^ |
| AT5G23940.1 | *Arabidopsis thaliana* | DCR | Biosynthesis cutin | ^33^ |
| AT5G25390.2 | *Arabidopsis thaliana* | SHN3 | Regulation | ^34^ |
| AT5G25390.1 | *Arabidopsis thaliana* | SHN3 | Regulation | ^34^ |
| AT5G33370.1 | *Arabidopsis thaliana* | Homolog of CD1 | Biosynthesis cutin | ^6^ |
| AT5G33370.2 | *Arabidopsis thaliana* | Homolog of CD1 | Biosynthesis cutin | ^6^ |
| AT5G61590.1 | *Arabidopsis thaliana* | DEWAX | Regulation | ^1^ |
| AT5G62470.2 | *Arabidopsis thaliana* | MYB96 | Regulation | ^35^ |
| AT5G62470.1 | *Arabidopsis thaliana* | MYB96 | Regulation | ^35^ |
| AT4G33790.1 | *Arabidopsis thaliana* | CER4 | Biosynthesis | ^36^ |
| AT5G37300.1 | *Arabidopsis thaliana* | WSD1 | Biosynthesis | ^37^ |
| AT5G57800.1 | *Arabidopsis thaliana* | CER3 | Biosynthesis | ^34^ |
| AF302098 | *Zea mays* | GL8a | Biosynthesis | ^38^ |
| AF527771 | *Zea mays* | GL8b | Biosynthesis | ^38^ |
| GRMZM2G026643_T02 | *Zea mays* | OCL1 | Regulation | ^39^ |
| GRMZM2G026643_T01 | *Zea mays* | OCL1 | Regulation | ^39^ |
| Medtr5G014400 | *Medicago truncatula* | IRG1 | Regulation | ^40^ |
| Medtr5G062700 | *Medicago truncatula* | WXP1 | Biosynthesis | ^41^ |
| NP_001304751.1 | *Solanum lycopersicum* | CER6 | Biosynthesis | ^42^ |
| Os02G31140 | *Oryza sativa* | CFL1 | Regulation | ^19^ |
| Solyc01G091630 | *Solanum lycopersicum* | CD2 | Biosynthesis cutin | ^43^ |
| Solyc11G006250 | *Solanum lycopersicum* | CD1 | Biosynthesis cutin | ^44^ |
| AMQ48725.1 | *Hordeum vulgare* | Eceriferum-c | Biosynthesis | ^45^ |
| AMQ48723.1 | *Hordeum vulgare* | Eceriferum-q | Biosynthesis | ^45^ |
| AMQ48724.1 | *Hordeum vulgare* | Eceriferum-u | Biosynthesis | ^45^ |
| TaDMP_protein | *Triticum aestivum* | NA | Biosynthesis | ^46^ |
| TaDMH_protein | *Triticum aestivum* | NA | Biosynthesis | ^46^ |
| TaDMC_protein | *Triticum aestivum* | NA | Biosynthesis | ^46^ |
| NB-Arc-like_1_protein | *Triticum aestivum* | NA | Biosynthesis | ^46^ |
| NB-Arc-like_2_protein | *Triticum aestivum* | NA | Biosynthesis | ^46^ |
| DUF4220_protein | *Triticum aestivum* | NA | Biosynthesis | ^46^ |
| PKS-1.1_protein | *Triticum aestivum* | NA | Biosynthesis | ^46^ |
| PKS-1.2_protein | *Triticum aestivum* | NA | Biosynthesis | ^46^ |
| P450-1.1_protein | *Triticum aestivum* | NA | Biosynthesis | ^46^ |
| Hyd-1.1_protein | *Triticum aestivum* | NA | Biosynthesis | ^46^ |
| Hyd-1.2(pseudogene)_protein | *Triticum aestivum* | NA | Biosynthesis | ^46^ |
| HlyIII-1.1_protein | *Triticum aestivum* | NA | Biosynthesis | ^46^ |
| P450-1.2(pseudogene)_protein | *Triticum aestivum* | NA | Biosynthesis | ^46^ |
| Hyd-1.3_protein | *Triticum aestivum* | NA | Biosynthesis | ^46^ |
| HlyIII-1.2(partial_protein) | *Triticum aestivum* | NA | Biosynthesis | ^46^ |
| PKS-2.1_protein | *Triticum aestivum* | NA | Biosynthesis | ^46^ |
| P450-2.1_protein | *Triticum aestivum* | NA | Biosynthesis | ^46^ |
| P450-3.1_protein | *Triticum aestivum* | NA | Biosynthesis | ^46^ |
| PKS-2.2(partial)_protein | *Triticum aestivum* | NA | Biosynthesis | ^46^ |
| P450-2.2_protein | *Triticum aestivum* | NA | Biosynthesis | ^46^ |
| P450-3.2(pseudogene)_protein | *Triticum aestivum* | NA | Biosynthesis | ^46^ |
| PKS-3_protein | *Triticum aestivum* | NA | Biosynthesis | ^46^ |
| Hyd-2_protein | *Triticum aestivum* | NA | Biosynthesis | ^46^ |
| Waxestersynthase(WES)_protein | *Triticum aestivum* | NA | Biosynthesis | ^46^ |
| Ank_PGG_protein | *Triticum aestivum* | NA | Biosynthesis | ^46^ |
| HQ266579 | *Solanum lycopersicum* | SlTTS1 | Regulation | ^47^ |
| HQ266580 | *Solanum lycopersicum* | SlTTS2 | Regulation | ^47^ |

References:

1. Go, Y. S., Kim, H., Kim, H. J. and Suh, M. C. 2014, Arabidopsis cuticular wax biosynthesis is negatively regulated by the DEWAX gene encoding an AP2/ERF-Type transcription factor. *Plant Cell*, **26**, 1666-1680.

2. Li-Beisson, Y., Pollard, M., Sauveplane, V., Pinot, F., Ohlrogge, J. and Beisson, F. 2009, Nanoridges that characterize the surface morphology of flowers require the synthesis of cutin polyester. *Proc. Natl Acad. Sci. USA*, **106**, 22008-22013.

3. Bernard, A., Domergue, F., Pascal, S., et al. 2012, Reconstitution of plant alkane biosynthesis in yeast demonstrates that *Arabidopsis* ECERIFERUM1 and ECERIFERUM3 are core components of a very-long-chain alkane synthesis complex. *Plant Cell*, **24**, 3106-3118.

4. Bonaventure, G., Salas, J. J., Pollard, M. R. and Ohlrogge, J. B. 2003, Disruption of the FATB gene in *Arabidopsis* demonstrates an essential role of saturated fatty acids in plant growth. *Plant Cell*, **15**, 1020-1033.

5. Lam, P., Zhao, L., McFarlane, H. E., et al. 2012, RDR1 and SGS3, components of RNA-mediated gene silencing, are required for the regulation of cuticular wax biosynthesis in developing inflorescence stems of *Arabidopsis*. *Plant Physiol.*, **159**, 1385-1395.

6. Shi, J. X., Malitsky, S., De Oliveira, S., et al. 2011, SHINE transcription factors act redundantly to pattern the archetypal surface of *Arabidopsis* flower organs. *PLoS Genet.*, **7**, e1001388.

7. Bird, D., Beisson, F., Brigham, A., et al. 2007, Characterization of *Arabidopsis* ABCG11/WBC11, an ATP binding cassette (ABC) transporter that is required for cuticular lipid secretion. *Plant J.*, **52**, 485-498.

8. Kim, H., Lee, S. B., Kim, H. J., Min, M. K., Hwang, I. and Suh, M. C. 2012, Characterization of glycosylphosphatidylinositol-anchored lipid transfer protein 2 (LTPG2) and overlapping function between LTPG/LTPG1 and LTPG2 in cuticular wax export or accumulation in *Arabidopsis thaliana*. *Plant Cell Physiol.*, **53**, 1391-1403.

9. Lü, S., Song, T., Kosma, D. K., Parsons, E. P., Rowland, O. and Jenks, M. A. 2009, *Arabidopsis* CER8 encodes LONG-CHAIN ACYL-COA SYNTHETASE 1 (LACS1) that has overlapping functions with LACS2 in plant wax and cutin synthesis. *Plant J.*, **59**, 553-564.

10. Panikashvili, D., Shi, J. X., Schreiber, L. and Aharoni, A. 2011, The *Arabidopsis* ABCG13 transporter is required for flower cuticle secretion and patterning of the petal epidermis. *New Phytol.*, **190**, 113-124.

11. Pighin, J. A., Zheng, H., Balakshin, L. J., et al. 2004, Plant cuticular lipid export requires an ABC transporter. *Science*, **306**, 702-704.

12. Greer, S., Wen, M., Bird, D., et al. 2007, The cytochrome P450 enzyme CYP96A15 is the midchain alkane hydroxylase responsible for formation of secondary alcohols and ketones in stem cuticular wax of *Arabidopsis*. *Plant Physiol.*, **145**, 653-667.

13. Pulsifer, I. P., Kluge, S. and Rowland, O. 2012, *Arabidopsis* LONG-CHAIN ACYL-COA SYNTHETASE 1 (LACS1), LACS2, and LACS3 facilitate fatty acid uptake in yeast. *Plant Physiol Biochem.*, **51**, 31-39.

14. Kurdyukov, S., Faust, A., Nawrath, C., et al. 2006, The epidermis-specific extracellular BODYGUARD controls cuticle development and morphogenesis in *Arabidopsis*. *Plant Cell*, **18**, 321-339.

15. Beaudoin, F., Wu, X., Li, F., et al. 2009, Functional characterization of the *Arabidopsis* β-Ketoacyl-Coenzyme a reductase candidates of the fatty acid elongase. *Plant Physiol.*, **150**, 1174-1191.

16. Fiebig, A., Mayfield, J. A., Miley, N. L., Chau, S., Fischer, R. L. and Preuss, D. 2000, Alterations in *CER6*, a gene identical to *CUT1*, differentially affect long-chain lipid content on the surface of pollen and stems. *Plant Cell*, **12**, 2001-2008.

17. Voisin, D., Nawrath, C., Kurdyukov, S., et al. 2009, Dissection of the complex phenotype in cuticular mutants of *Arabidopsis* reveals a role of SERRATE as a mediator. *PLoS Genet.*, **5**, e1000703.

18. Bessire, M., Borel, S., Fabre, G., et al. 2011, A member of the PLEIOTROPIC DRUG RESISTANCE family of ATP binding cassette transporters is required for the formation of a functional cuticle in *Arabidopsis*. *Plant Cell*, **23**, 1958-1970.

19. Wu, R., Li, S., He, S., et al. 2011, CFL1, a WW domain protein, regulates cuticle development by modulating the function of HDG1, a class IV homeodomain transcription factor, in rice and *Arabidopsis*. *Plant Cell*, **23**, 3392-3411.

20. Wellesen, K., Durst, F., Pinot, F., et al. 2001, Functional analysis of the LACERATA gene of Arabidopsis provides evidence for different roles of fatty acid ω-hydroxylation in development. *Proc. Natl Acad. Sci. USA*, **98**, 9694-9699.

21. Oshima, Y., Shikata, M., Koyama, T., Ohtsubo, N., Mitsuda, N. and Ohme-Takagi, M. 2013, MIXTA-like transcription factors and WAX INDUCER1/SHINE1 coordinately regulate cuticle development in *Arabidopsis* and *Torenia fournieri*. *Plant Cell*, **25**, 1609-1624.

22. Pascal, S., Bernard, A., Sorel, M., et al. 2013, The *Arabidopsis* *cer26* mutant, like the *cer2* mutant, is specifically affected in the very long chain fatty acid elongation process. *Plant J.*, **73**, 733-746.

23. Raffaele, S., Vailleau, F., Léger, A., et al. 2008, A MYB transcription factor regulates very-long-chain fatty acid biosynthesis for activation of the hypersensitive cell death response in *Arabidopsis*. *Plant Cell*, **20**, 752-767.

24. Lee, S. B., Kim, H. U. and Suh, M. C. 2016, MYB94 and MYB96 additively activate cuticular wax biosynthesis in *Arabidopsis*. *Plant Cell Physiol.*, **57**, 2300-2311.

25. Zheng, H., Rowland, O. and Kunst, L. 2005, Disruptions of the *Arabidopsis* Enoyl-CoA reductase gene reveal an essential role for very-long-chain fatty acid synthesis in cell expansion during plant morphogenesis. *Plant Cell*, **17**, 1467-1481.

26. Doan, T. T. P., Carlsson, A. S., Hamberg, M., Bülow, L., Stymne, S. and Olsson, P. 2009, Functional expression of five Arabidopsis fatty acyl-CoA reductase genes in *Escherichia coli*. *J. Plant Physiol.*, **166**, 787-796.

27. Hooker, T. S., Lam, P., Zheng, H. and Kunst, L. 2007, A core subunit of the RNA-processing/degrading exosome specifically influences cuticular wax biosynthesis in *Arabidopsis*. *Plant Cell*, **19**, 904-913.

28. Jessen, D., Olbrich, A., Knüfer, J., et al. 2011, Combined activity of LACS1 and LACS4 is required for proper pollen coat formation in *Arabidopsis*. *Plant J.*, **68**, 715-726.

29. Haslam, T. M., Mañas-Fernández, A., Zhao, L. and Kunst, L. 2012, *Arabidopsis* ECERIFERUM2 is a component of the fatty acid elongation machinery required for fatty acid extension to exceptional lengths. *Plant Physiol.*, **160**, 1164-1174.

30. Cominelli, E., Sala, T., Calvi, D., Gusmaroli, G. and Tonelli, C. 2008, Over-expression of the *Arabidopsis* AtMYB41 gene alters cell expansion and leaf surface permeability. *Plant J.*, **53**, 53-64.

31. Lü, S., Zhao, H., Des Marais, D. L., et al. 2012, *Arabidopsis* ECERIFERUM9 involvement in cuticle formation and maintenance of plant water status. *Plant Physiol.*, **159**, 930-944.

32. Bach, L., Michaelson, L. V., Haslam, R., et al. 2008, The very-long-chain hydroxy fatty acyl-CoA dehydratase PASTICCINO2 is essential and limiting for plant development. *Proc. Natl Acad. Sci. USA*, **105**, 14727-14731.

33. Rani, S. H., Krishna, T. H. A., Saha, S., Negi, A. S. and Rajasekharan, R. 2010, Defective in cuticular ridges (DCR) of *Arabidopsis thaliana*, a gene associated with surface cutin formation, encodes a soluble diacylglycerol acyltransferase. *J. Biol. Chem.*, **285**, 38337-38347.

34. Aharoni, A., Dixit, S., Jetter, R., Thoenes, E., van Arkel, G. and Pereira, A. 2004, The SHINE clade of AP2 domain transcription factors activates wax biosynthesis, alters cuticle properties, and confers drought tolerance when overexpressed in *Arabidopsis*. *Plant Cell*, **16**, 2463-2480.

35. Seo, P. J., Lee, S. B., Suh, M. C., Park, M.-J., Go, Y. S. and Park, C.-M. 2011, The MYB96 transcription factor regulates cuticular wax biosynthesis under drought conditions in *Arabidopsis*. *Plant Cell*, **23**, 1138-1152.

36. Rowland, O., Zheng, H., Hepworth, S. R., Lam, P., Jetter, R. and Kunst, L. 2006, *CER4* encodes an alcohol-forming fatty Acyl-Coenzyme A reductase involved in cuticular wax production in *Arabidopsis*. *Plant Physiol.*, **142**, 866-877.

37. Li, F., Wu, X., Lam, P., et al. 2008, Identification of the wax ester synthase/Acyl-Coenzyme A:diacylglycerol acyltransferase WSD1 required for stem wax ester biosynthesis in *Arabidopsis*. *Plant Physiol.*, **148**, 97-107.

38. Dietrich, C. R., Perera, M. A. D. N., D. Yandeau-Nelson, M., Meeley, R. B., Nikolau, B. J. and Schnable, P. S. 2005, Characterization of two GL8 paralogs reveals that the 3-ketoacyl reductase component of fatty acid elongase is essential for maize (*Zea mays L.*) development. *Plant J.*, **42**, 844-861.

39. Javelle, M., Vernoud, V., Depège-Fargeix, N., et al. 2010, Overexpression of the epidermis-specific homeodomain-leucine zipper IV transcription factor OUTER CELL LAYER1 in Maize identifies target genes involved in lipid metabolism and cuticle biosynthesis. *Plant Physiol.*, **154**, 273-286.

40. Chen, J., Yu, J., Ge, L., et al. 2010, Control of dissected leaf morphology by a Cys(2)His(2) zinc finger transcription factor in the model legume Medicago truncatula. *Proc. Natl Acad. Sci. USA*, **107**, 10754-10759.

41. Zhang, J.-Y., Broeckling, C. D., Blancaflor, E. B., Sledge, M. K., Sumner, L. W. and Wang, Z.-Y. 2005, Overexpression of WXP1, a putative Medicago truncatula AP2 domain-containing transcription factor gene, increases cuticular wax accumulation and enhances drought tolerance in transgenic alfalfa (Medicago sativa). *Plant J.*, **42**, 689-707.

42. Smirnova, A., Leide, J. and Riederer, M. 2013, Deficiency in a very-long-chain fatty acid β-ketoacyl-coenzyme a synthase of tomato impairs microgametogenesis and causes floral organ fusion. *Plant Physiol.*, **161**, 196-209.

43. Nadakuduti, S. S., Pollard, M., Kosma, D. K., Allen, C., Ohlrogge, J. B. and Barry, C. S. 2012, Pleiotropic phenotypes of the *sticky peel* mutant provide new insight into the role of *CUTIN DEFICIENT2* in epidermal cell function in tomato. *Plant Physiol.*, **159**, 945-960.

44. Yeats, T. H., Martin, L. B. B., Viart, H. M. F., et al. 2012, The identification of cutin synthase: formation of the plant polyester cutin. *Nat Chem Biol.*, **8**, 609-611.

45. Schneider, L. M., Adamski, N. M., Christensen, C. E., et al. 2016, The *Cer-cqu* gene cluster determines three key players in a β-diketone synthase polyketide pathway synthesizing aliphatics in epicuticular waxes. *J. Exp. Bot.* , **67**, 2715-2730.

46. Hen-Avivi, S., Savin, O., Racovita, R., et al. 2016, A metabolic gene cluster in the wheat *W1* and the barley *Cer-cqu* loci determines β-diketone biosynthesis and glaucousness. *Plant Cell*.

47. Wang, Z., Guhling, O., Yao, R., et al. 2011, Two oxidosqualene cyclases responsible for biosynthesis of tomato fruit cuticular triterpenoids. *Plant Physiol.*, **155**, 540-552.

**Supplementary Table 6.** Blueberry unigenes annotated by Waxybase proteins.

| Blueberry unigene hits | Gene ID | Species | Gene name |
| --- | --- | --- | --- |
| VACC_DN106858_c0_g1 | AT1G68530.1 | *Arabidopsis* | CER6 |
| VACC_DN153512_c0_g1 | AT5G33370.2 | *Arabidopsis* | Homolog of CD1 |
| VACC_DN15466_c0_g1 | AT3G01140.1 | *Arabidopsis* | MYB106 |
| VACC_DN25114_c0_g2 | AT1G15360.1 | *Arabidopsis* | WIN1/ SHN1 |
| VACC_DN39517_c0_g1 | AT2G26910.1 | *Arabidopsis* | ABCG32 |
| VACC_DN40891_c0_g1 | AT1G17840.1 | *Arabidopsis* | ABCG11/WBC11 |
| VACC_DN55048_c0_g1 | AT4G28110.1 | *Arabidopsis* | MYB41 |
| VACC_DN64879_c0_g1 | AT2G26910.1 | *Arabidopsis* | ABCG32 |
| VACC_DN65089_c0_g1 | AT2G26910.1 | *Arabidopsis* | ABCG32 |
| VACC_DN66305_c0_g1 | AT2G26910.1 | *Arabidopsis* | ABCG32 |
| VACC_DN6750_c0_g1 | AT1G72970.1 | *Arabidopsis* | HTH |
| VACC_DN68218_c1_g1 | Solyc11G006250 | *Solanum lycopersicum* | CD1 |
| VACC_DN74126_c0_g1 | AT1G68530.2 | *Arabidopsis* | CER6 |
| VACC_DN74126_c1_g1 | AT1G68530.1 | *Arabidopsis* | CER6 |
| VACC_DN76322_c1_g1 | AT4G23850.1 | *Arabidopsis* | LACS4 |
| VACC_DN76944_c0_g1 | AT2G26910.1 | *Arabidopsis* | ABCG32 |
| VACC_DN77134_c1_g1 | AT2G26910.1 | *Arabidopsis* | ABCG32 |
| VACC_DN77656_c1_g1 | AT5G33370.1 | *Arabidopsis* | Homolog of CD1 |
| VACC_DN78690_c2_g1 | AT1G72970.1 | *Arabidopsis* | HTH |
| VACC_DN79042_c6_g5 | AT2G26910.1 | *Arabidopsis* | ABCG32 |
| VACC_DN80252_c1_g1 | AT5G10480.1 | *Arabidopsis* | PAS2 |
| VACC_DN80508_c0_g1 | AT1G60810.1 | *Arabidopsis* | ACLA2 |
| VACC_DN81809_c2_g1 | AT1G08510.1 | *Arabidopsis* | FATB |
| VACC_DN81954_c0_g1 | HQ266579.1 | *Solanum lycopersicum* | SlTTS1 |
| VACC_DN83432_c0_g1 | AT1G01120.1 | *Arabidopsis* | KCS1 |
| VACC_DN83687_c0_g1 | AT4G28110.1 | *Arabidopsis* | MYB41 |
| VACC_DN84231_c2_g1 | AT1G49340.2 | *Arabidopsis* | LACS2 |
| VACC_DN84460_c3_g1 | AT1G14790.1 | *Arabidopsis* | RDR1 |
| VACC_DN84802_c1_g1 | NP_001304751.1 | *Solanum lycopersicum* | CER6 |
| VACC_DN85095_c1_g1 | Medtr5G062700 | *Medicago. truncatula* | WXP1 |
| VACC_DN85289_c0_g1 | AT3G55360.1 | *Arabidopsis* | CER10 |
| VACC_DN85289_c0_g2 | AT3G55360.1 | *Arabidopsis* | CER10 |
| VACC_DN85817_c4_g1 | AT2G45970.1 | *Arabidopsis* | LCR |
| VACC_DN86271_c1_g1 | AT4G24140.1 | *Arabidopsis* | BDG3 |
| VACC_DN87402_c1_g3 | Solyc01G091630 | *Solanum lycopersicum* | CD2 |
| VACC_DN87468_c1_g3 | AF527771 | Maize | GL8b |
| VACC_DN87757_c4_g3 | AT1G17840.1 | *Arabidopsis* | ABCG11/WBC11 |
| VACC_DN88022_c7_g1 | AT3G60500.3 | *Arabidopsis* | CER7 |
| VACC_DN8822_c1_g1 | AT2G45970.1 | *Arabidopsis* | LCR |
| VACC_DN88741_c0_g1 | AT1G04220.1 | *Arabidopsis* | KCS2 |
| VACC_DN89506_c0_g1 | AT2G47240.2 | *Arabidopsis* | LACS1/CER8 |
| VACC_DN89797_c1_g1 | AT1G04220.1 | *Arabidopsis* | KCS2 |
| VACC_DN89797_c1_g2 | AT1G04220.1 | *Arabidopsis* | KCS2 |
| VACC_DN89809_c6_g1 | AT2G45970.1 | *Arabidopsis* | LCR |
| VACC_DN89809_c8_g1 | AT2G45970.1 | *Arabidopsis* | LCR |
| VACC_DN89809_c9_g2 | AT2G45970.1 | *Arabidopsis* | LCR |
| VACC_DN90163_c1_g2 | AT2G26250.1 | *Arabidopsis* | FDH |
| VACC_DN90599_c3_g2 | AT4G34100.2 | *Arabidopsis* | CER9 |
| VACC_DN90599_c3_g3 | AT4G34100.2 | *Arabidopsis* | CER9 |
| VACC_DN90599_c3_g4 | AT4G34100.1 | *Arabidopsis* | CER9 |
| VACC_DN90694_c6_g3 | AT2G26910.1 | *Arabidopsis* | ABCG32 |
| VACC_DN90761_c3_g1 | AT1G49340.2 | *Arabidopsis* | LACS2 |
| VACC_DN90778_c2_g1 | AT2G38110.1 | *Arabidopsis* | GPAT6 |
| VACC_DN91087_c3_g1 | AT1G60810.1 | *Arabidopsis* | ACLA2 |
| VACC_DN91087_c4_g1 | AT1G60810.1 | *Arabidopsis* | ACLA2 |
| VACC_DN91087_c6_g1 | AT1G60810.1 | *Arabidopsis* | ACLA2 |
| VACC_DN91148_c4_g1 | AT4G23850.1 | *Arabidopsis* | LACS4 |
| VACC_DN91219_c2_g1 | HQ266579.1 | *Solanum lycopersicum* | SlTTS1 |
| VACC_DN91372_c3_g1 | AT1G04220.1 | *Arabidopsis* | KCS2 |
| VACC_DN92143_c3_g1 | AT2G26910.1 | *Arabidopsis* | ABCG32 |
| VACC_DN92143_c3_g2 | AT2G26910.1 | *Arabidopsis* | ABCG32 |
| VACC_DN92218_c3_g1 | AT1G51500.1 | *Arabidopsis* | ABCG12/CER5 |
| VACC_DN92386_c4_g2 | NP_001304751.1 | *Solanum lycopersicum* | CER6 |
| VACC_DN97829_c0_g1 | AT2G26910.1 | *Arabidopsis* | ABCG32 |
| VACC_DN112059_c0_g1 | G3F-11051-MONOMER | *Solanum lycopersicum* | FAR |
| VACC_DN12094_c0_g2 | G33-6511-MONOMER | *Vitis vinifera* | CER1 |
| VACC_DN15514_c0_g1 | G3F-11051-MONOMER | *Solanum lycopersicum* | FAR |
| VACC_DN156414_c0_g1 | G3F-9203-MONOMER | *Solanum lycopersicum* | CER1 |
| VACC_DN53137_c0_g1 | G33-6501-MONOMER | *Vitis vinifera* | CER1 |
| VACC_DN57709_c0_g1 | G3F-11051-MONOMER | *Solanum lycopersicum* | FAR |
| VACC_DN81895_c7_g3 | G2Z-9840-MONOMER | *Manihot esculenta esculenta* | FAR |
| VACC_DN84992_c2_g2 | G3F-10871-MONOMER | *Solanum lycopersicum* | CER1 |
| VACC_DN85527_c1_g1 | G3M-10042-MONOMER | *Solanum tuberosum* | Cytochrome P450 |
| VACC_DN85527_c3_g2 | G3F-13206-MONOMER | *Solanum lycopersicum* | Cytochrome P450 |
| VACC_DN89021_c0_g1 | G37-12288-MONOMER | *Populus trichocarpa* | CER3 |
| VACC_DN90116_c0_g1 | G3F-10817-MONOMER | *Solanum lycopersicum* | FAR |
| VACC_DN90116_c2_g1 | G33-7309-MONOMER | *Vitis vinifera* | FAR |
| VACC_DN90116_c2_g2 | G33-7309-MONOMER | *Vitis vinifera* | FAR |
| VACC_DN91870_c4_g1 | G33-9975-MONOMER | *Vitis vinifera* | CER3 |

**Supplementary Table 7.** Predicted DEGs in ‘Nocturne’ x T 300 population.

**Supplementary Table 8.** Predicted DEGs in ‘Nocturne’ x US 1212 population.

**Supplementary Table 9.** Real-time qPCR data for selected DEGs.

**Supplementary Table 10.** Real-time qPCR data for 17 major genes involved in wax accumulation selected from literature.

**Data in Supplementary Tables 7-10 are provided in separate xls files, Additional files 2-5.**
